# New Quinolinone O-GlcNAc Transferase Inhibitors Based on Fragment Growth

**DOI:** 10.3389/fchem.2021.666122

**Published:** 2021-04-14

**Authors:** Matjaž Weiss, Elena M. Loi, Maša Sterle, Cyril Balsollier, Tihomir Tomašič, Roland J. Pieters, Martina Gobec, Marko Anderluh

**Affiliations:** ^1^The Chair of Pharmaceutical Chemistry, Faculty of Pharmacy, University of Ljubljana, Ljubljana, Slovenia; ^2^Department of Chemical Biology and Drug Discovery, Utrecht Institute for Pharmaceutical Sciences, Utrecht, Netherlands; ^3^The Chair of Clinical Biochemistry, Faculty of Pharmacy, University of Ljubljana, Ljubljana, Slovenia

**Keywords:** O-GlcNAc, O-GlcNAc transferase, protein glycosylation, fragments growth, molecular docking

## Abstract

O-GlcNAcylation is an important post-translational and metabolic process in cells that must be carefully regulated. O-GlcNAc transferase (OGT) is ubiquitously present in cells and is the only enzyme that catalyzes the transfer of O-GlcNAc to proteins. OGT is a promising target in various pathologies such as cancer, immune system diseases, or nervous impairment. In our previous work we identified the 2-oxo-1,2-dihydroquinoline-4-carboxamide derivatives as promising compounds by a fragment-based drug design approach. Herein, we report the extension of this first series with several new fragments. As the most potent fragment, we identified **3b** with an IC_50_ value of 116.0 μM. If compared with the most potent inhibitor of the first series, **F20** (IC_50_ = 117.6 μM), we can conclude that the new fragments did not improve OGT inhibition remarkably. Therefore, **F20** was used as the basis for the design of a series of compounds with the elongation toward the O-GlcNAc binding pocket as the free carboxylate allows easy conjugation. Compound **6b** with an IC_50_ value of 144.5 μM showed the most potent OGT inhibition among the elongated compounds, but it loses inhibition potency when compared to the UDP mimetic **F20**. We therefore assume that the binding of the compounds in the O-GlcNAc binding pocket is likely not crucial for OGT inhibition. Furthermore, evaluation of the compounds with two different assays revealed that some inhibitors most likely interfere with the commercially available UDP-Glo™ glycosyltransferase assay, leading to false positive results. This observation calls for caution, when evaluating UDP mimetic as OGT inhibitors with the UDP-Glo™ glycosyltransferase assay, as misinterpretations can occur.

## Introduction

O-GlcNAcylation is an essential post-translational modification known to modify more than a thousand of proteins in the human nucleus and cytosol (Torres and Hart, 1984; Haltiwanger et al., 1990; Ma and Hart, 2014). O-β-*N*-acetylglucosaminyl transferase (OGT) is the only enzyme that catalyzes the transfer of *N*-acetylglucosamine (O-GlcNAc) from UDP-GlcNAc to serine and threonine residues of proteins. On the other hand, the delicate equilibrium of O-GlcNAcylation status is balanced by O-GlcNAcase (OGA), which cleaves O-GlcNAc residues (Haltiwanger et al., 1990; Kreppel et al., 1997). In a physiological environment, the process of O-GlcNAcylation is dynamic and is regulated not only by the activity and expression level of both enzymes, but also by the availability of UDP-GlcNAc nutrients (Haltiwanger et al., 1992) and the post-translational modification of target proteins (e.g., crosstalk with kinases) (Kreppel et al., 1997; Ryu and Do, 2011). Previous work has shown that impaired O-GlcNAcylation of proteins modulates their functions (Yang et al., 2006), consequently having an impact on the cell signaling (Butkinaree et al., 2010; Dias et al., 2012), the cell cycle (Liu and Li, 2018), transcription (Butkinaree et al., 2010), and also epigenetic processes (Sakabe et al., 2010). This highlights that O-GlcNAc homeostasis is crucial for normal cell activity (Yang et al., 2006; Butkinaree et al., 2010). Dysregulated O-GlcNAcylation has been observed in diabetes (Hart et al., 2011), cancer (Ma and Vosseller, 2013; Ferrer et al., 2016), cardiovascular diseases (Marsh et al., 2014), Alzheimer's disease (Yuzwa and Vocadlo, 2014), and immune system diseases (Abramowitz and Hanover, 2018). However, the precise role of O-GlcNAcylation in many biological systems is not well understood, in part because a potent, selective, and permeable OGT inhibitor is needed to study OGT function in a cellular or *in vivo* environment. While several OGT inhibitors are already known (Ortiz-Meoz et al., 2015; Trapannone et al., 2016; Martin et al., 2018), they have shortcomings such as a lack of specificity and limited cell permeability (Trapannone et al., 2016).

**Figure 1:**
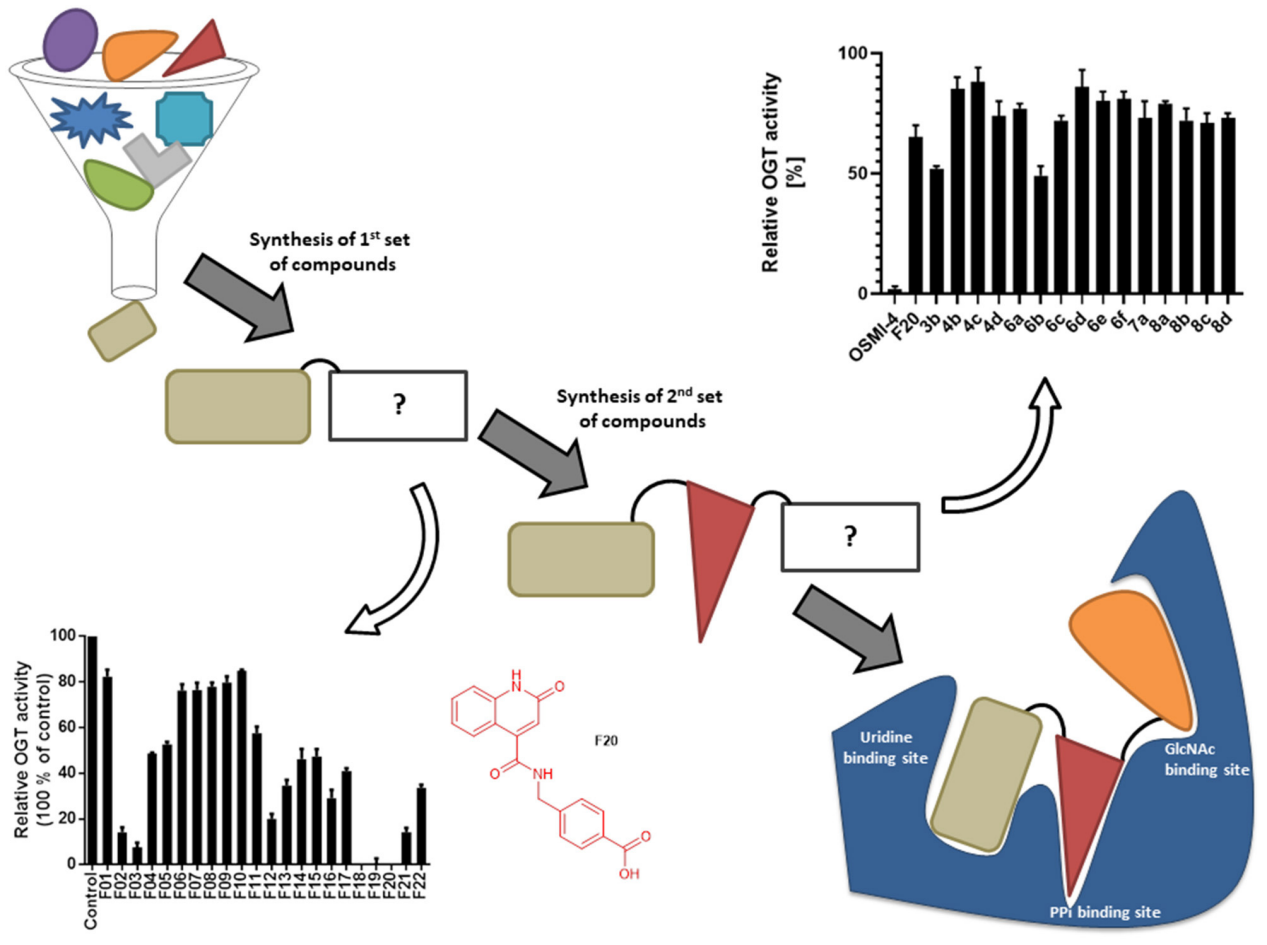
Schematic depiction of fragment screening and workflow of the fragment-based design approach; synthesis and evaluation of 1^st^ set of 22 compounds (evaluation at 1 mM by UDP-Glo™ assay) and 2^nd^ set of 15 compounds (evaluation at 100 μM by fluorescence activity assay). 2^nd^ set includes compounds based on **F20** elongated with various amines.

Recently, we have designed and published first OGT inhibitors with a 2-hydroxyquinoline-4-carboxamide scaffold by a fragment-based drug design (FBDD) approach based on structure-based virtual screening (Zhang et al., 2018). Meanwhile, Martin et al. published an OGT crystal structure with quinolone-based compounds of OSMI family inhibitors. This confirmed our earlier predictions that the hydrogen bond donor-acceptor pair is crucial for positioning the quinolinone fragment in the UDP-binding site of OGT (Martin et al., 2018; Zhang et al., 2018). The promising results of the 2-hydroxyquinoline-4-carboxamide-based compounds and confirmed interactions of the quinolinone-based inhibitors provide the rationale for additional elongation of the most potent compound (**F20**) in a linear manner (Figure 1). It is based on a 2-hydroxyquinoline-4-carboxylic acid scaffold that mimics the uridine moiety and is elongated with 4-(aminomethyl)benzoic acid toward the GlcNAc-binding site (Zhang et al., 2018). Compounds were evaluated using the commercially available UDP-Glo™ glycosyltransferase assay, which measures luminescence in a luciferase reaction after released UDP is converted to ATP (Promega, 2020).

Lately, the reliability of these results has been questioned and a new direct fluorescence activity assay reported by the Vocadlo group has been put forward. In this work, we describe the design and synthesis of a new focused library of OGT inhibitors obtained by further growing of the **F20** fragment. To avoid misinterpretation in the identification of new OGT inhibitors, we assayed all compounds using the Vocadlo and the UDP-Glo™ glycosyltransferase assay (Vocadlo et al., 2020). By using both of the aforementioned assays, we determined their inhibition constants in the micromolar range. Importantly, we emphasize that caution should be used when interpreting results obtained with the UDP-Glo™ glycosyltransferase assay, as it may yield false-positive results.

## Materials and Methods

### Chemistry

All reagents and solvents were commercially available and used without further purification. Water used for isolations was purified. Column chromatography was carried out on silica gel 60 Merck 0.040–0.063 mm and preparative thin-layer chromatography (TLC) on silica gel plates F254 from Merck. ^1^H NMR and ^13^C NMR spectra were recorded using Bruker Avance III 400 spectrometer operating at 400 Hz for ^1^H and 101 Hz for ^13^C, using TMS as internal standard and DMSO-*d*_6_ as solvent. The chemical shifts (δ values) and coupling constants *(J* values) are given in ppm and hertz (Hz), respectively. High resolution mass spectra were recorded at the Exactive ^TM^ Plus Orbitrap Mass Spectrometer and VG-Analytical Autospec Q spectrometer. The mass spectra were recorded on Advion expression L mass spectrometer. IR spectra were recorded on FT-IR Thermo Nicolet spectrometer, using attenuated total reflectance technique. Melting points were obtained using a Kofler hot-stage microscope.

### UDP-Glo™ Glycosyltransferase Assay

This assay evaluates O-GlcNAcylation through monitoring UDP formation in glycosyltransferase reactions by luminescence. Briefly, OGT reactions were carried out in a 12.5 μL final volume in the well of a with 96-well microplate containing 0.1 mM UDP-GlcNAc, 200 nM purified full-length OGT, 100 μM RBL-2 peptide in OGT reaction buffer (25 mM Tris-HCl, pH 7.5; 1 mM DTT; 12.5 mM MgCl_2_). All the reactions are made in triplicate over the same plate. Reactions were always incubated at 37°C for 2 h. Afterwards, each reaction is completed by UDP-Glo Detection Reagent to a 1:1 ratio. After incubation at room temperature for 1 h, the luminescence was recorded with a POLARstar® Omega microplate reader (BMG LABTECH) and Synergie H4 Hybrid reader (BIOTEK). The data were normalized and plotted with GraphPad prism 8.2.1 software.

### Fluorescent Activity Assay

The fluorescent activity assay was performed as published (Vocadlo et al., 2020). OGT reactions were carried out in a 25 μL final volume containing 2.8 μM glycosyl donor BFL-UDP-GlcNAc, 200 nM purified full-length OGT, 9.2 μM glycosyl acceptor HCF-1 Serine in OGT reaction buffer (1 × PBS pH 7.4, 1 mM DTT, 12.5 mM MgCl_2_). Reactions were incubated at room temperature for 1 h in the presence of different concentrations of inhibitor (the inhibitors were pre-incubated with OGT for at least 5 min). The reactions were then stopped by a mix of UDP at a final concentration of 2 mM and a solution of Nanolink magnetic streptavidin beads (2 μL of stock solution per reaction). After incubation at room temperature for 30 min, the beads were immobilized on a magnetic surface and washed thoroughly with PBS-tween 0.01%. Finally, the beads were resuspended in PBS-tween 0.01% and transferred to a microplate for endpoint fluorescence measurement. Fluorescence was read at Ex/Em 485/530 with a POLARstar® Omega microplate reader (BMG LABTECH) and Synergie H4 Hybrid reader (BIOTEK). The data were normalized and plotted with GraphPad prism 8.2.1 software. The concentration of the inhibitor where the residual activity of the enzyme is 50% (IC_50_) was calculated using a nonlinear regression-based fitting of inhibition curves using the (inhibitor) vs. response-variable slope (four parameters).

### Molecular Modeling

For docking with FRED software (OEDOCKING 3.3.1.2, OpenEye Scientific Software, Inc., Santa Fe, NM, USA; www.eyesopen.com), OGT-binding site (PDB entry: 4GYY) was prepared using MAKE RECEPTOR (Release 3.3.1.2, OpenEye Scientific Software, Inc., Santa Fe, NM, USA; www.eyesopen.com) (McGann, 2011, 2012; Kelley et al., 2015; OpenEye, 2020). The grid box around the ligand UDP-5S-GlcNAc bound in the OGT crystal structure was generated automatically and was not adjusted. This resulted in a box with the following dimensions: 21.67 Å ^*^18.33 Å ^*^ 21.33 Å and the volume of 8474 Å^3^. For “Cavity detection” slow and effective “Molecular” method was used for detection of binding sites. Inner and outer contours of the grid box were also calculated automatically using “Balanced” settings for “Site Shape Potential” calculation. The inner contours were disabled. Ala896 was defined as hydrogen bond donor and acceptor constraint for the docking calculations. The ligands were prepared by OMEGA (Release 3.3.1.2, OpenEye Scientific Software, Inc., Santa Fe, NM, USA; www.eyesopen.com) and were then docked to the prepared binding site of OGT using FRED (default settings). The resulting file was saved as SDF format and edited with PyMOL (The PyMOL Molecular Graphics System, Version 1.5.0.3 Schrödinger, LLC).

## Results and Discussion

**Figure 2:**
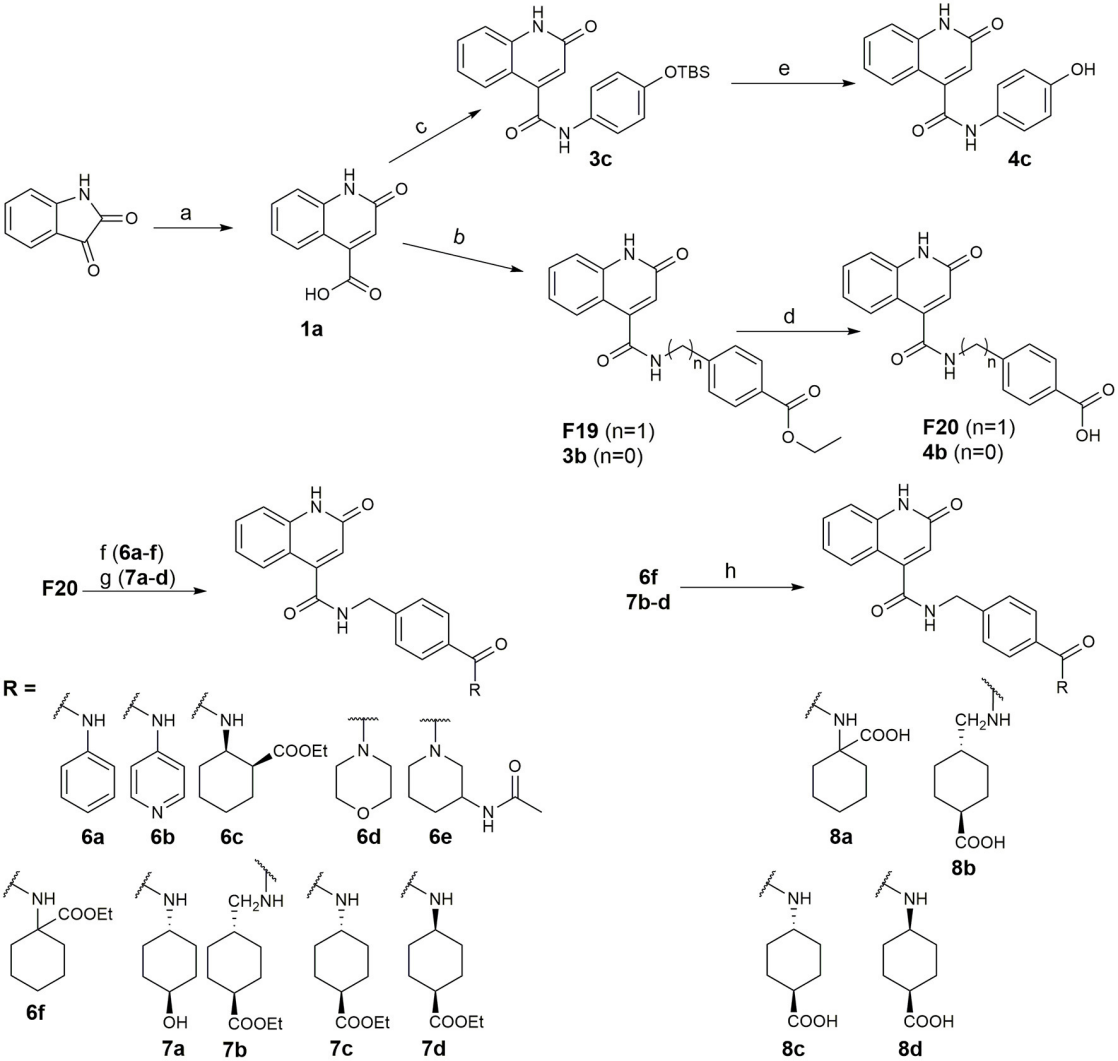
Reagents and conditions (a) acetic acid, malonic acid, reflux, overnight; (b) and (f) corresponding amine, DIPEA, TBTU, DMF, RT, overnight; (c) ethyl chloroformate, TEA, DMF, corresponding amine, RT, overnight; (d) and (h) NaOH, EtOH, RT, overnight; (e) TBAF, THF, RT, overnight; (g) corresponding amine, HOBt, EDC, Et_3_N, DMF, RT, overnight.

**Figure 3:**
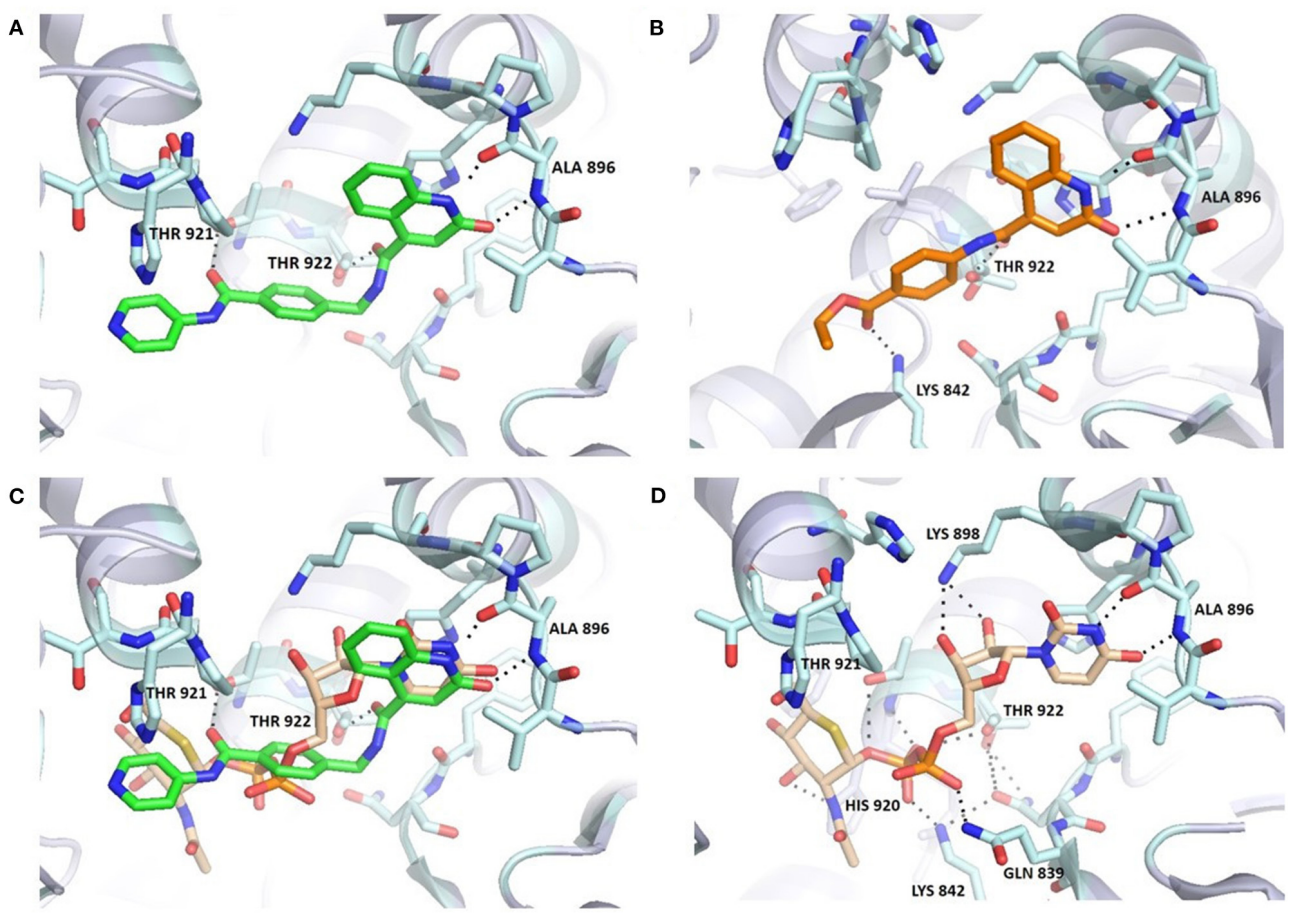
Comparison of **6b (A**,**C)** and UDP-5S-GlcNAc **(C**,**D)** binding mode in the OGT binding site (PDB entry: 4GYY); predicted binding pose for **3b (B)**. The ligand and the neighboring protein side-chains are shown as stick models, colored according to the chemical atom type (blue, N; red, O; orange, S; green, Cl). Hydrogen bonds are indicated by black dotted lines. Thr922 is doubled due to static disorder.

Recently, we reported the first quinolinone-based OGT inhibitors, targeting the uridine diphosphate (UDP)-binding pocket. Evaluation by UDP-Glo™ glycosyltransferase assay revealed that the most potent fragment **F20** (IC_50_ = 117.6 μM; Supplementary Material Figure 1A) of the first series can be elongated without the loss of potency. By molecular docking of the same fragment, we revealed that the OGT active site offers enough space for the fragment growth toward the O-GlcNAc-binding pocket. To further explore this finding, fragment growth of **F20** (Figure 1) was performed by conjugating its free carboxylic acid with different aromatic and aliphatic amines *via* amide bond (Figure 2). Our aim was to improve interactions by elongation toward the O-GlcNAc-binding pocket, and consequently, improve inhibitory potency. Borodkin et al. found that in the series of peptide-O-GlcNAc-conjugated OGT inhibitors, the O-GlcNAc moiety decreased binding affinity (Borodkin et al., 2014). We assumed that this loss was due to desolvation penalties of the O-GlcNAc moiety since this outcompetes newly formed hydrogen bonds so that no gain in affinity is observed. However, the O-GlcNAc moiety does form some hydrogen bonds with OGT (Lazarus et al., 2012). Consequently, we have sought suitable saccharide replacements by introducing more lipophilic rings devoid of full –OH-decorated pyranose ring, while still having some hydrogen bond donors/acceptors to target-specific OGT residues (such as His920), as depicted in Figure 3. To investigate the impact of the linker length and flexibility on inhibitory potency, shorter fragments were introduced by removing a methylene group (compounds **3** and **4**).

Compounds were screened at 240 μM using the UDP-Glo™ glycosyltransferase assay (Promega, 2020) and the new direct fluorescence activity assay (Vocadlo et al., 2020). As shown in Table 1, the residual activities of some compounds (**4c**, **6a**, **6f**) varied significantly between assays, indicating that these compounds are likely to interfere with UDP-Glo™ assay. Namely, the latter is a coupled assay that involves not only the GlcNAcylation reaction, but also the conversion of UDP to ATP by the Uridine/Cytidine Monophosphate Kinase (CMK) (Zegzouti et al., 2014), and the final luciferase reaction in which luminescence occurs upon ATP consumption. Therefore, there is a substantial chance that one of the above reaction steps is perturbed. Since our compounds are UDP mimetics there is a great possibility that they could interfere with the UDP-to-ATP conversion on which the assay is based. Considering that our compounds are UDP mimetics and the enzyme included in assay is CMK, which catalyzes the transfer of a phosphoryl group from UDP to ADP, the UDP-Glo™ assay should be carefully evaluated prior to evaluation of UDP mimetics that are very likely to bind CMK and inhibit the phosphate transfer. Otherwise, we found the UDP-Glo™ assay simple and fast to perform, but according to the results obtained, the assay is susceptible to interference by UDP mimetics. On the other hand, the fluorescence activity assay of Vocadlo et al. directly measures the GlcNAcylation level by using a modified GlcNAc donor molecule and is not a coupled assay, which in our opinion makes it a preferred option for OGT inhibitor screening.

**Table 1:** Fragments and compounds inhibition potency against OGT.

**Frag**.	** 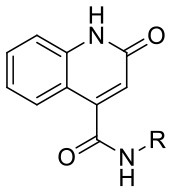 **	OGT residual activity [%]	
		**Fluorescence activity assay**	**UDP-Glo™ assay**
F20	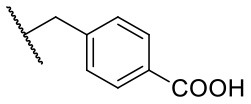	41 ± 5	60 ± 1
3b	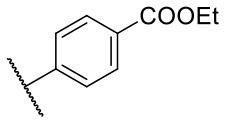	44 ± 5	48 ± 2
4b	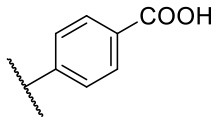	82 ± 7	94 ± 2
4c	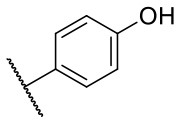	65 ± 9	9 ± 0
Comp.	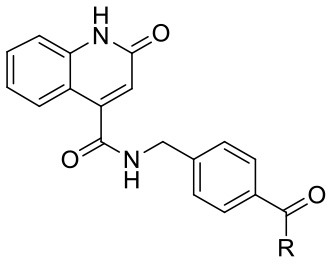	OGT residual activity [%]	
		Fluorescence activity assay	UDP-Glo™ assay
6a	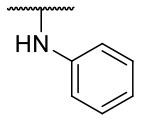	77 ± 5	29 ± 1
6b	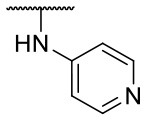	21 ± 1	68 ± 0
6c	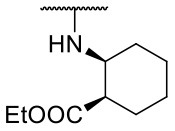	88 ± 4	83 ± 1
6d	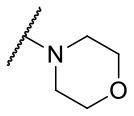	65 ± 1	75 ± 0
6e	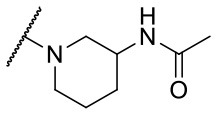	72 ± 0	95 ± 2
6f	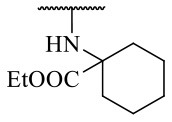	77 ± 8	40 ± 0
7a	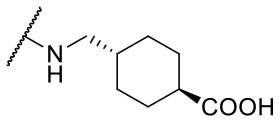	63 ± 3	64 ± 1
8a	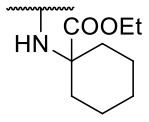	64 ± 0	85 ± 0
8b	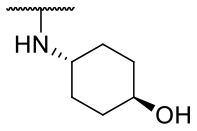	64 ± 4	69 ± 1
8c	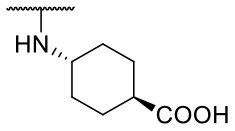	79 ± 0	77 ± 1
8d	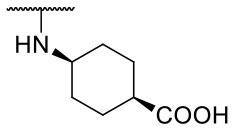	71 ± 4	100 ± 0
OSMI-4^*^		0 ± 0	0 ± 0

Results are presented as mean ± SD from two independent experiments, each performed in triplicate. Compounds were tested at 240 μM;

* *OSMI-4 was tested at 50 μM*.

Based on the one-point screening, we identified compounds **3b** and **6b** as the most potent inhibitors; therefore, the IC_50_ values were determined for these compounds. Due to the difficulties with UDP-Glo™ assay described above, we determined the inhibition constants using the fluorescence activity assay. For fragment **3b**, the IC_50_ was determined at 116.0 μM (Supplementary Material Figure 1B). Compound **6b** extended with the 4-pyridyl fragment, is the most potent compound from the second series with an IC_50_ value of 144.5 μM (Supplementary Material Figure 1C). Unfortunately, no notable improvement in inhibitory potency was achieved after elongation of the starting fragment **F20**.

Molecular docking of **6b** using FRED software predicted hydrogen bonding with Ala896, Thr921, and Thr922 (Figure 3). The orientation of the quinolinone ring of **6b** and its hydrogen bond acceptor-donor formation with Ala896 mimics the binding mode of the uracil moiety of UDP-5S-GlcNAc (Figure 3C). Additionally, the carbonyl groups of the amides featured in the inhibitor are predicted to form two hydrogen bonds with Thr921 and Thr922, which are also important for UDP binding. The length of the synthesized compounds is similar to the UDP-5S-GlcNAc, but the predicted binding pose shows a difference in the orientation of the pyridine ring of **6b** in comparison with GlcNAc. There is also an additional hydrogen bond between GlcNAc –OH at position 3 and His920. An overlay in Figure 3C shows the space for potential modification of the compound to form hydrogen bonds with Lys898 and Lys842, amino acids residues crucial for the OGT catalytic activity. The predicted binding pose of **6b** is similar to that of **F20**, which also does not form hydrogen bonds with Lys898 and Lys842. In contrast, the predicted binding mode of **3b**, the shorter analog of **F20**, shows an additional interaction with Lys842 and a loss of the hydrogen bond with Thr921 (Figure 3B). According to the assay results of **F20** and its shorter analog **3b**, the difference in interactions with amino acid residues in the binding pocket did not affect potency of the compounds. In light of the above discoveries, optimization of the GlcNAc mimetic is necessary to assure additional interactions in the O-GlcNAc-binding pocket.

## Conclusion

In conclusion, a new set of OGT inhibitors was designed and synthesized. We identified compounds that block the monosaccharide donor site of OGT by a combination of virtual screening and fragment growth synthesis. The most potent fragment from the first set was used for elongation with various amines. A set of elongated compounds was then evaluated at the OGT enzyme by direct fluorescence assay and by UDP-Glo™ assay. The latter was found to be a potentially problematic assay for the screening of UDP mimetics due to possible interference with the CMK enzyme that converts UDP to ATP and giving false positives. Compared to the first set of fragments, the binding affinities were not improved and further optimization is required. The most active compound in the enzymatic assay was the compound **6b** with an IC_50_ value of 144.5 μM. We additionally identified the new potential fragment **3b** with an IC_50_ value of 116.0 μM. The binding affinities of the compounds are still in the higher micromolar range and further studies will be needed to develop more potent OGT inhibitors.

## Data Availability Statement

Publicly available datasets were analyzed in this study. This data can be found here: https://www.rcsb.org/structure/4GYY/b.

## Author Contributions

MW wrote the manuscript with input and comments from RP, MG, TT, and MA. MW, MS, and EL synthesized all compounds. CB performed *in vitro* assays. EL performed molecular docking. MA supervised the chemical synthesis. RP, MG, and MA supervised the performance of *in vitro* assays. MA and TT conceived the idea. All authors have read and agreed to the published version of the manuscript.

## Conflict of Interest

The authors declare that the research was conducted in the absence of any commercial or financial relationships that could be construed as a potential conflict of interest.
