# Supplementary material for: New Quinolinone O-GlcNAc Transferase Inhibitors Based on Fragment Growth
Source: Front Chem. 2021 Apr 14;9:666122. doi: 10.3389/fchem.2021.666122 (PMC8079942; doi:10.3389/fchem.2021.666122)
Supplement: Supplementary file 1 [file Data_Sheet_1.PDF]

Supporting information for the article

New quinolinone O-GlcNAc transferase inhibitors based on fragment growth

Matjaž Weiss[1], Elena M. Loi[1,3], Maša Sterle[1], Cyril Balsollier[1,3], Tihomir Tomašič[1], Roland J. Pieters[3], Martina Gobec[2*] and Marko Anderluh[1*]

[1]The Chair of Pharmaceutical Chemistry, University of Ljubljana, Faculty of Pharmacy, Ljubljana, Slovenia

[2]The Chair of Clinical Biochemistry, University of Ljubljana, Faculty of Pharmacy, Ljubljana, Slovenia

[3]Department of Chemical Biology & Drug Discovery, Utrecht Institute for Pharmaceutical Sciences, Utrecht, the Netherlands

Supplementary Table S1. Fragments and compounds OGT inhibition at 100 µM

Compound	OGT activity [%]
	Fluorescence activity assay
**F20**	**65 ± 5**
**3b**	**52 ± 1**
**4b**	**85 ± 5**
**4c**	**74 ± 6**
**6a**	**77 ± 2**
**6b**	**49 ± 4**
**6c**	**72 ± 2**
**6d**	**86 ± 7**
**6e**	**80 ± 4**
**6f**	**81 ± 3**
**7a**	**73 ± 7**
**8a**	**79 ± 1**
**8b**	**72 ± 5**
**8c**	**71 ± 4**
**8d**	**73 ± 2**
**OSMI-4***	**2 ± 1**

Compounds were tested at 100 µM; mean ± SD from two independent experiments, each performed in triplicate.
*OSMI-4 was tested at 50 µM.

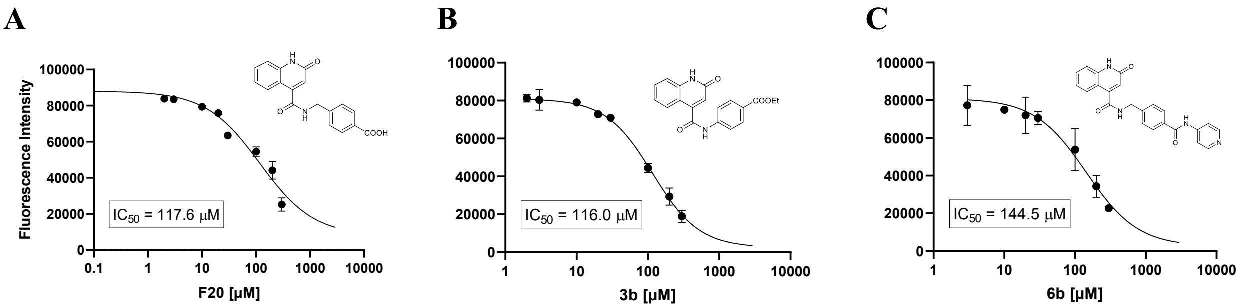

Supplementary Figure S1. IC_{50} curves of **F20 (A)**, **3b (B)** and **6b (C)**. The results are presented as fluorescence intensity (mean ± SD) from two independent experiments, each performed in triplicate.

Experimental details and characterization data of new compounds

Procedures for the synthesis of fragments 1-4

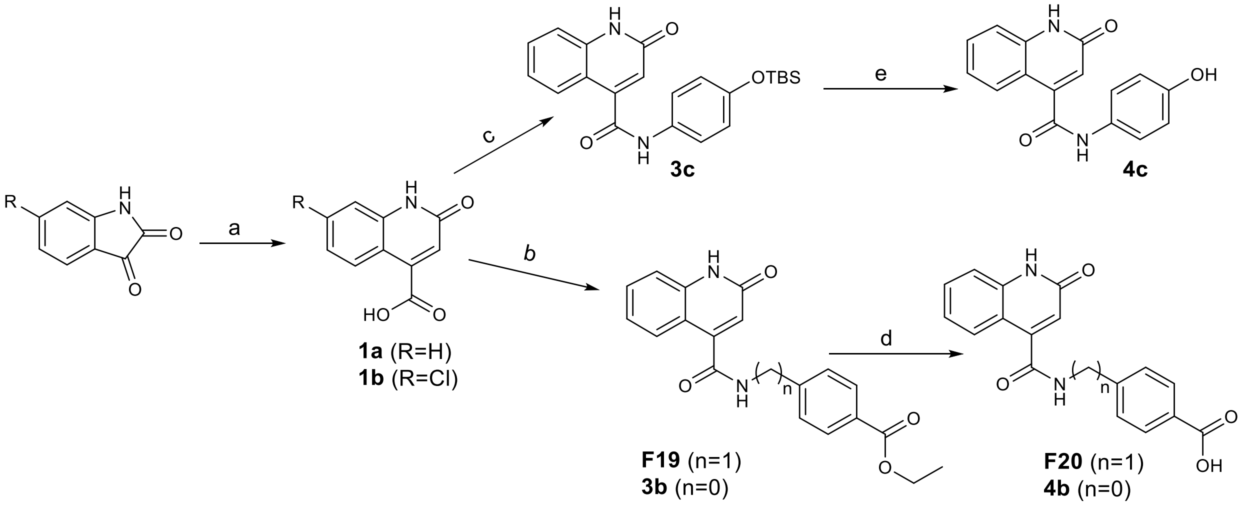

Supplementary Figure S2. Procedures for the synthesis of fragments **1**-**4**. Reagents and conditions: a) acetic acid, malonic acid, reflux, overnight; b) corresponding amine, DIPEA, TBTU, DMF, RT, overnight; c) ethyl chloroformate, TEA, DMF, corresponding amine, RT, overnight; d) NaOH, EtOH, RT, overnight; e) TBAF, THF, RT, overnight.

Fragments **1** were synthesized according to established literature procedure (Dulla et al., 2012). Indoline-2,3-dione (11.99 g; 81.50 mmol) or 6-chloro-indoline-2,3-dione (5.00 g; 27.6 mmol) was added to a solution of malonic acid (3 eq) in 250 mL acetic acid. The mixture was refluxed and stirred overnight under an argon atmosphere. The solution was concentrated under reduced pressure; the crude mixture was resuspended in water (300 mL) and filtered. Solid residue was resuspended in saturated $NaHCO_3$ (400 mL) and filtered. The liquid fraction was acidified with concentrated HCl to pH = 1-2. Precipitate was filtered and recrystallized from 30 mL EtOH to obtain solid fragment **1**.

2-Oxo-1,2-dihydroquinoline-4-carboxylic acid (**1a**). White solid (15.42 g; 77.8%); m.p.: 248.2-250.2 °C; ^{1}**H NMR** (400 MHz, DMSO-d_6): δ (ppm) 12.06 (s, 1H, Ar-NH-CO) 8.10 (dd, 1H, J = 8.3, 1.4 Hz, Ar-H), 7.51 (ddd, 1H, J = 8.3, 7.0, 1.4 Hz, Ar-H), 7.38 (dd, 1H, J = 8.3, 1.4 Hz, Ar-H), 7.19 (ddd, 1H, J = 8.3, 7.0, 1.4 Hz, Ar-H), 6.77 (s, 1H, Ar-H); ^{13}**C NMR** (101 MHz, DMSO-d_6): δ (ppm) 166.78 (COOH-Ar), 160.99 (Ar-NH-CO), 141.15, 139.39, 130.85, 126.09, 123.38, 122.22, 115.74, 115.72 (Ar-C); **HRMS** (ESI-): m/s calcd for $C_{10}H_6NO_3$ ([M-H]$^-$): 188.0348, found 188.0350; **IR** (ATR): ν (cm^{-1}) 2989, 2883, 2577, 1712, 1652, 1544, 1509, 1477, 1433, 1385, 1354, 1323, 1279, 1258, 1229, 1184, 1158, 1041, 1006, 940, 874, 794, 759, 744, 712, 654, 635, 622, 549, 526.

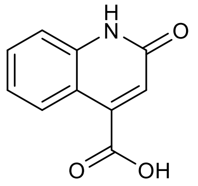

7-Chloro-2-oxo-1,2-dihydroquinoline-4-carboxylic acid (**1b**). Pink solid (3.20 g; 53.2%); ^{1}**H NMR** (400 MHz, DMSO-d_6): δ (ppm) 11.57 (s, 1H, Ar-NH-CO) 8.24 (d, 1H, J = 8.7 Hz, Ar-H), 7.28 (d, 1H, J = 2.1 Hz, Ar-H), 7.13 (dd, 1H, J = 8.7, 2.1 Hz, Ar-H), 6.34 (s, 1H, Ar-H).

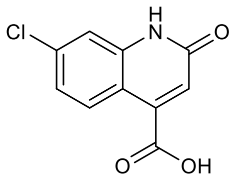

4-(Ethoxycarbonyl)phenyl)methanaminium chloride (**2**)**.** Fragment **2** was synthesized according to

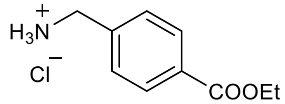

established slightly modified literature procedure (Chhun and Schmitzer, 2011). 4-(Aminomethyl)benzoic acid (6.00 g; 39.69 mmol) was dissolved in 200 mL anhydrous EtOH. The mixture was cooled down to 0 °C on ice bath under an argon atmosphere and $SOCl_2$ (6.60 mL; 90.99 mmol) was added dropwise. The reaction mixture was stirred at 50 °C for approximately 24 h. The solution was concentrated under reduced pressure. Trituration with Et_2O afforded white crystals (**2**). (8.16 g; 95.3%); m.p.: 222.2-224.8 °C; **^{1}H NMR** (400 MHz, DMSO-d_6): δ (ppm) 8.65 (bs, 3H, NH_3^+), 7.98 (d, 2H, *J* = 8.5 Hz, Ar-H), 7.65 (d, 2H, *J* = 8.5 Hz, Ar-H), 4.32 (q, 2H, *J* = 7.1 Hz, $\underline{CH_2}CH_3$), 4.10 (q, 2H, *J* = 5.7 Hz, Ar-CH_2), 1.32 (t, 3H, *J* = 7.1 Hz, $CH_2\underline{CH_3}$); **IR** (KBr): ν (cm^{-1}) 2963, 2888, 2756, 2690, 2582, 1715, 1594, 1480, 1468, 1447, 1368, 1216, 1188, 1125, 1106, 1076, 1024, 972, 879, 858, 759, 701, 524.

Ethyl 4-((2-oxo-1,2-dihydroquinoline-4-carboxamido)methyl)benzoate (**F19**). Fragment **F19** was

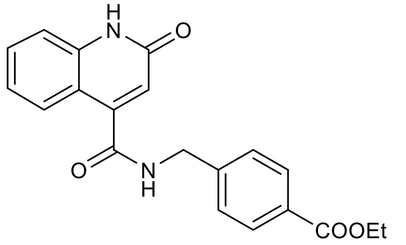

synthesized according to established literature procedure (Zhang et al., 2018). Fragment **1a** (5.00 g; 26.43 mmol) and **2** (5.70 g; 26.43 mmol) were dissolved in anhydrous DMF (200 mL) and DIPEA (4.60 mL; 26.43 mmol) under an argon atmosphere. After 10 minutes of stirring at room temperature TBTU (11.03 g; 34.36 mmol) was added. The reaction mixture was stirred at room temperature for 20 h. The precipitate formed in the flask was filtered and the solution was concentrated under reduced pressure. The residue was dissolved in DCM (200 mL) and washed with water (2 × 50 mL), saturated $NaHCO_3$ (4 × 50 mL) and brine (50 mL). The combined organic layers were dried over Na_2SO_4, filtered and concentrated under reduced pressure to yield white solid fragment (**F19**). (6.18 g; 66.8%); m.p.: 269.4-271.4 °C; **^{1}H NMR** (400 MHz, DMSO-d_6): δ (ppm) 12.04 (s, 1H, Ar-NH-CO) 9.49 (t, 1H, *J* = 6.0 Hz, $\underline{NH}$-CH_2), 7.97-7.95 (m, 2H, Ar-H), 7.68 (dd, 1H, *J* = 8.3, 1.2 Hz, Ar-H), 7.56–7.49 (m, 3H, Ar-H), 7.41 (dd, 1H, *J* = 8.3, 1.2 Hz, Ar-H), 7.19 (ddd, 1H, *J* = 8.3, 7.1, 1.2 Hz, Ar-H), 6.59 (d, 1H, *J* = 1.2 Hz, Ar-H), 4.56 (d, 2H, *J* = 6.0 Hz, $NH\underline{CH_2}$), 4.31 (q, 2H, *J* = 7.1 Hz, $\underline{CH_2}CH_3$), 1.32 (t, 3H, *J* = 7.1 Hz, $CH_2\underline{CH_3}$); **^{13}C NMR** (101 MHz, DMSO-d_6): δ (ppm) 165.91 (Ar-COO-), 165.56 (Ar-$\underline{CO}$-NH-CH_2), 161.19 (Ar-NH-$\underline{CO}$), 145.85, 144.46, 139.21, 130.86, 129.33, 128.55, 127.38, 125.80, 122.09, 119.90, 116.06, 115.66 (14 × C-Ar), 60.65 ($\underline{CH_2}$-CH_3), 42.14 (NH-$\underline{CH_2}$), 14.16 ($\underline{CH_3}$-CH_2); **HRMS** (ESI-): m/z calcd for $C_{20}H_{17}N_2O_4$ ($[M-H]^-$) 349.1188, found 349.1183; **IR** (ATR): ν (cm^{-1}) 3283, 2987, 2828, 2737, 1710, 1664, 1639, 1611, 1542, 1479, 1439, 1423, 1415, 1276, 1263, 1191, 1177, 1160, 1127, 1109, 1036, 1021, 951, 912, 881, 843, 797, 772, 764, 752, 746, 714, 690, 654, 642, 561, 522, 510.

Ethyl 4-(2-oxo-1,2-dihydroquinoline-4-carboxamido)benzoate (**3b**). Fragment **1a** (400 mg; 2.12

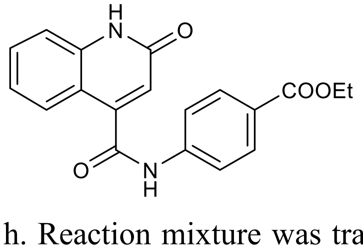

mmol) were dissolved in 40 mL of toluene under an argon atmosphere. $SOCl_2$ (180 µL; 2.33 mmol) and anhydrous DMF (6 drops) were added dropwise. After 2 h at 40 °C solution was concentrated under reduced pressure and again dissolved in toluene. Ethyl 4-aminobenzoate (347 mg; 2.12 mmol) and DIPEA (1.20 mL; 6.75 mmol) were added. The reaction mixture was stirred at 40 °C under an argon atmosphere for 24 h. Reaction mixture was transferred to a separating funnel and washed with 40 mL of water. Water phase was separated and filtered. Formed particles were dried to obtain white solid fragment (**3b**). (256 mg; 36.1%); m.p.: >300 °C; **^{1}H NMR** (400 MHz, DMSO-d_6): δ (ppm) 12.05 (s, 1H, Ar-NH-CO), 11.08 (s, 1H, Ar-CO-NH-Ar), 8.02 – 7.98 (m, 2H, Ar-H), 7.93 – 7.89 (m, 2H, Ar-H), 7.72 (dd, 1H, *J* = 8.1, 1.0 Hz, Ar-H,), 7.57 (ddd, 1H, *J* = 8.4, 7.2, 1.3 Hz, Ar-H), 7.40 (dd, 1H, *J* = 8.1, 1.0 Hz , Ar-H), 7.22 (ddd, 1H, *J* = 8.4, 7.2, 1.3 Hz, Ar-H), 6.76 (d, 1H, *J* = 1.6 Hz, Ar-H), 4.31 (q, 2H, *J* = 7.1

Hz, -CH_2-CH_3), 1.33 (t, 3H, *J* = 7.1 Hz, -CH_2-CH_3); **^{13}C NMR** (101 MHz, DMSO-*d_6*): δ (ppm) 165.25 (Ar-CO-NH-Ar), 164.69 (Ar-COO-), 161.15 (Ar-NH-CO-), 145.45, 142.85, 139.27, 131.02, 130.22, 125.62, 125.13, 122.77, 120.30, 199.38, 115.80, 115.76 (14 × C-Ar), 60.55 (-CH_2-CH_3), 14.19 (-CH_2-CH_3); **HRMS** (ESI-): m/z calcd for $C_{19}H_{15}N_2O_4$ ($[M-H]^-$) 337.1183, found 337.1173; **IR** (ATR): ν (cm^{-1}) 3279, 2992, 2846, 2737, 1704, 1650, 1606, 1594, 1548, 1522, 1469, 1435, 1412, 1393, 1366, 1313, 1263, 1174, , 1156, 1103, 1040, 1021, 982, 915, 860, 840, 791, 763, 751, 711, 688, 651, 586, 558.

N-(4-((*tert*-Butyldimethylsilyl)oxy)phenyl)-2-oxo-1,2-dihydroquinoline-4-carboxamide (**3c**). A

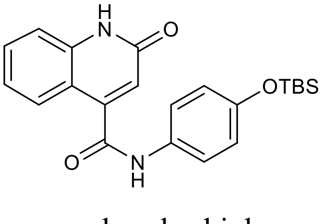

solution of **1a** (378 mg, 2.01 mmol) and triethylamine (0.30 mL; 2.20 mmol) in 3 mL of DMF was cooled to 0 °C under an argon atmosphere and ethyl chloroformate (0.21 mL; 2.20 mmol) was added dropwise. The mixture was stirred at room temperature for 30 min, then cooled again to 0 °C before adding 4-(*tert*-butyldimethylsiloxy)aniline (447 mg; 2.00 mmol). The reaction was stirred at room temperature overnight. DMF was removed under high vacuum and the residue was partitioned between ethyl acetate and 0.1 M HCl. The organic layer was washed with $NaHCO_3$ and brine, then dried over Na_2SO_4 and concentrated under vacuum. The crude product was purified by flash chromatography eluting with 3% MeOH/DCM to obtain a white solid (137 mg, 17.4%); m.p.: >300 °C; **^{1}H NMR** (400 MHz, DMSO-*d_6*): δ (ppm) 11.81 (s, 1H, Ar-NH-CO), 10.42 (s, 1H, Ar-CO-NH-Ar), 7.58 – 7.50 (m, 1H, Ar-H), 7.46 – 7.39 (m, 2H, Ar-H), 7.37 (ddd, 1H, *J* = 8.2, 7.1, 1.9 Hz, Ar-H), 7.23 – 7.14 (m, 1H, Ar-H), 7.02 (ddd, 1H, *J* = 8.2, 7.1, 1.9 Hz, Ar-H), 6.71 – 6.61 (m, 2H, Ar-H), 6.48 (d, 1H, *J* = 1.9 Hz, Ar-H), 0.77 (s, 9H, $C(CH_3)_3$), 0.01 (s, 6H, Si-$(CH_3)_2$).

General procedure for synthesis fragment F20 and 4b.

Fragment **F19** or **3b** was dissolved in 150 mL EtOH and 1 M NaOH (6 eq). The reaction mixture was stirred overnight at room temperature. 20 mL of water was added and EtOH was removed under reduced pressure. The pH in water solution was adjusted to 1-2 and formed precipitate was filtered and dried to obtain solid particles.

4-((2-Oxo-1,2-dihydroquinoline-4-carboxamido)methyl)benzoic acid (**F20**) (Hogg et al., 2017).

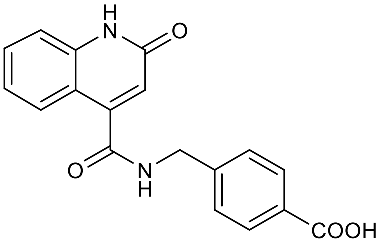

White solid (5.27 g; 94.18%); m.p.: > 300°C; **^{1}H NMR** (400 MHz, DMSO-*d_6*): δ (ppm) 11.99 (s, 1H, Ar-NH-CO), 9.40 (t, 1H, *J* = 6.0 Hz, NH-CH_2), 7.94 (d, 2H, *J* = 8.2 Hz, Ar-H), 7.69 (dd, 1H, *J* = 8.3, 1.3, Ar-H), 7.54 (ddd, 1H, *J* = 8.3, 7.1, 1.3 Hz, Ar-H), 7.48 (d, 2H, *J* = 8.2 Hz, Ar-H), 7.36 (d, 1H, *J* = 8.3 Hz, , Ar-H), 7.20 (ddd, 1H, *J* = 8.3, 7.1, 1.3 Hz, Ar-H), 6.60 (s, 1H, Ar-H), 4.56 (d, 2H, *J* = 6.0 Hz, NHCH_2); **^{13}C NMR** (101 MHz, DMSO-*d_6*): δ (ppm) 170.06 (COOH-Ar), 165.76 (Ar-CO-NH-CH_2), 161.24 (Ar-NH-CO), 146.05, 139.72, 139.24, 138.20, 130.78, 129.20, 126.14, 125.77, 122.01, 119.76, 116.14, 115.72 (14 × C-Ar), 42.24 (CH_2-NHCO); **HRMS** (ESI-): m/z calcd for $C_{18}H_{13}N_2O_4$ ($[M-H]^-$) 321.0875, found 321.0872; **IR** (ATR): ν (cm^{-1}) 3239, 2958, 2640, 2517, 1714, 1633, 1536, 1418, 1259, 1203, 1176, 1156, 1113, 1038, 1022, 902, 845, 774, 744, 686, 657, 626, 556, 509.

4-(2-Oxo-1,2-dihydroquinoline-4-carboxamido)benzoic acid (**4b**). Brown solid (172 mg; 78.9%);

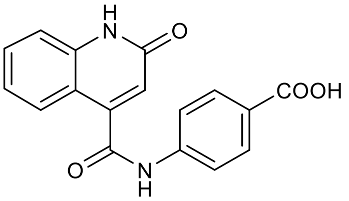

m.p.: > 300 °C; ^{1}**H NMR** (400 MHz, DMSO-d_6): δ (ppm) 12.84 (s, 1H, COOH), 12.05 (s, 1H, Ar-NH-CO), 11.05 (s, 1H, Ar-CO-NH-Ar), 7.98 – 7.95 (m, 2H, Ar-H), 7.89 – 7.85 (m, 2H, Ar-H), 7.71 (dd, 1H, *J* = 8.1, 1.0, Ar-H), 7.57 (ddd, 1H, *J* = 8.4, 7.2, 1.3, Ar-H), 7.39 (dd, 1H, *J* = 8.3, 0.6, Ar-H), 7.22 (ddd, 1H, *J* = 8.2, 7.2, 1.1, Ar-H), 6.75 (s, 1H, Ar-H); ^{13}**C NMR** (101 MHz, DMSO-d_6): δ (ppm) 167.83 (COOH-Ar), 165.64 (Ar-CO-NH-Ar), 162.16 (Ar-NH-CO), 146.52, 143.53, 140.27, 131.99, 2 × 131.3, 127.07, 126.62, 123.25, 121.24, 2 × 120.29, 116.83, 116.76 (14 × C-Ar); **HRMS** (ESI-): m/z calcd for $C_{17}H_{11}N_2O_4$ ([M-H]$^-$) 307.0724, found 307.0719; **IR** (ATR): ν (cm^{-1}) 3321, 3016, 2492, 1684, 1638, 1597, 1527, 1468, 1410, 1360, 1319, 1244, 1177, 1157, 1130, 1118, 1041, 1012, 946, 899, 853, 797, 768, 748, 721, 690, 674, 647, 551, 525.

2-Oxo-*N*-phenyl-1,2-dihydroquinoline-4-carboxamide (**4c**). Fragment **3c** (1 eq) was dissolved in 5

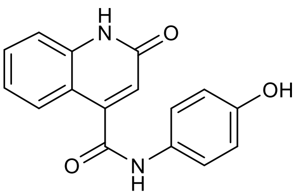

mL THF and cooled to 0 °C under an argon atmosphere for 10 minutes before TBAF (0.15 mL of 1 M solution in THF; 1.2 eq) was added dropwise. The reaction mixture was stirred overnight at room temperature. Solvent was removed under reduced pressure and residue was dissolved in ethyl acetate and washed with NH_4Cl and brine. The organic layer contained a precipitate that was filtered and dried to obtain **4c**. White solid (70.0 mg; 71.08%); m.p.: > 300 °C; ^{1}**H NMR** (400 MHz, DMSO-d_6) δ 11.99 (s, 1H, Ar-NH-CO), 10.48 (s, 1H, Ar-CO-NH-Ar), 9.35 (s, 1H, Ar-OH), 7.72 (d, 1H, *J* = 8.0 Hz, Ar-H), 7.60 – 7.49 (m, 3H, Ar-H), 7.37 (d, 1H, *J* = 8.3 Hz, Ar-H), 7.21 (dd, 1H, *J* = 7.9, 1.3 Hz, Ar-H), 6.79-6.72 (m, 2H, Ar-H), 6.64 (d, 1H, *J* = 1.9 Hz, Ar-H); ^{13}**C NMR** (101 MHz, DMSO-d_6): δ (ppm) 164.19 (Ar-CO-NH-Ar), 161.90 (Ar-NH-CO), 154.50, 146.76, 139.59, 131.44, 130.48, 126.23, 122.76, 2 × 122.30 , 120.06, 116.59, 116.20, 2 × 115.61; **HRMS** (ESI+): m/z calcd for $C_{16}H_{13}N_2O_3$ ([M+H]$^+$) 281.0921, found 281.0918; **IR** (ATR): ν (cm^{-1}) 3246, 3084, 2956, 2883, 2847, 1643, 1600, 1536, 1513, 1431, 1399, 1270, 1223, 1163, 1040, 912, 887, 779, 759,712.

General procedure for the synthesis of 5.

Compounds **5** were synthesized according to established slightly modified literature procedures. 0.20 g of the amine (1 eq) was dissolved in 10 mL anhydrous EtOH under an argon atmosphere. The reaction mixture was cooled down to 0 °C. The solution of $SOCl_2$ (3 eq) in 1 mL anhydrous EtOH was added dropwise. The reaction mixture was refluxed and stirred for 24 h. After completion of reaction solvent and $SOCl_2$ were evaporated to obtain compound **5**. Further purification was done by recrystallization from MeOH/Et_2O to afford pure **5**.

1-(Ethoxycarbonyl)cyclohexan-1-aminium chloride (**5a**). Pale yellow oil (0.22 g; 91.12 %); ^{1}**H NMR**

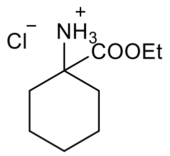

(400 MHz, DMSO-d$_6$): δ (ppm) 8.67 (s, 3H, NH_3^+), 4.19 (q, 2H, *J* = 6.2 Hz, <u>CH$_2$</u>CH$_3$), 1.92 (s, 2H, H-cyclohex.), 1.77 (s, 2H, H-cyclohex.), 1.67 (s, 2H, H-cyclohex.), 1.52 (s, 2H, H-cyclohex.), 1.40 (s, 2H, H- cyclohex.), 1.23 (t, 3H, *J* = 6.2 Hz, CH$_2$<u>CH$_3$</u>) (Hogg et al., 2017).

(*R*)-3-(Ethoxycarbonyl)piperidin-1-ium chloride (**5b**). White crystals (0.086 g; 28.7%); m.p.: 88.0 -

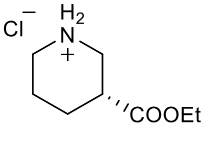

89.3 °C ^{1}**H NMR** (400 MHz, DMSO-d$_6$): δ (ppm) 9.23 (s, 2H, NH_2^+), 4.10 (q, 2H, *J* = 7.0 Hz, <u>CH$_2$</u>CH$_3$), 3.39–3.29 (m, 1H, H-pip.), 3.17–3.14 (m, 1H, H-pip.), 2.94–2.76 (m, 3H, H-pip.), 1.97 (dd, 1H, *J* = 13.5, 4.2 Hz, H-pip.), 1.75 (dq, 2H, *J* = 11.0, 7.2, 5.9 Hz, H-pip.), 1.61–1.52 (m, 1H, H-pip.), 1.19 (t, 3H, *J* = 7.0 Hz,

$CH_2\underline{CH_3}$); **IR** (ATR): ν (cm^{-1}) 3110, 2939, 2797, 2716, 2636, 2595, 2550, 2515, 2432, 1721, 1403, 1389, 1374, 1316, 1299, 1278, 1235, 1203, 1155, 1073, 1051, 1017, 927, 910, 881 (Dorogov et al., 2006).

(1*R*,4*R*)-4-(Ethoxycarbonyl)cyclohexyl)methanaminium chloride (**5c**). White crystals (1.306 g; 86.4 %); ^{1}**H NMR** (400 MHz, DMSO-d_6) δ (ppm) 8.12 (s, 3H, NH_3^+), 4.03 (q, 2H, *J* = 7.1 Hz, $\underline{CH_2}CH_3$), 2.61 (p, 2H, *J* = 5.8 Hz, $NH_3^+\underline{CH_2}$), 2.21 (tt, 1H, *J* = 12.1, 3.5 Hz, H-cyclohex.), 1.90 (dd, 2H, *J* = 13.7, 3.2 Hz, H-cyclohex.), 1.81 (dd, 2H, *J* = 13.6, 3.0 Hz, H-cyclohex.), 1.55 (m, 1H, H-cyclohex.), 1.34 – 1.21 (m, 1H, H-cyclohex.), 1.16 (t, 3H, *J* = 7.1 Hz, $CH_2\underline{CH_3}$), 1.03 – 0.90 (m, 1H, H-cyclohex.) (Shonberg et al., 2013).

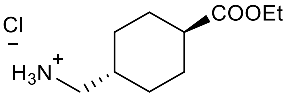

(1*S*,4*S*)-4-(Ethoxycarbonyl)cyclohexan-1-aminium cloride (**5d**). White crystals (286.0 mg; 98.6 %); ^{1}**H NMR** (400 MHz, DMSO-d_6) δ (ppm) 8.11 (s, 3H, NH_3^+), 4.08 (q, 2H, *J* = 7.0 Hz, $\underline{CH_2}CH_3$), 3.04 (s, 1H, H-cyclohex.), 2.63 – 2.54 (m, 1H, H-cyclohex.), 2.00 – 1.91 (m, 2H, H-cyclohex.), 1.83 – 1.75 (m, 2H, H-cyclohex.), 1.61 – 1.43 (m, 4H, H-cyclohex.), 1.19 (t, 3H, *J* = 7.0 Hz, $CH_2\underline{CH_3}$) (Curry et al., 2006).

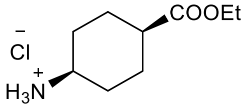

General procedure for the synthesis of 6.

Starting fragment **F20** (1 eq) was dissolved in 10 mL of anhydrous DMF under an argon atmosphere. DIPEA (4 eq) and TBTU (1.3 eq) were added to the solution and stirred for 10 minutes at room temperature. Then the amine (1.1 eq) was added and stirred overnight at room temperature. Solution was concentrated under reduced pressure and different ways of purification were performed depending on the properties of individual compound.

2-Oxo-*N*-(4-(phenylcarbamoyl)benzyl)-1,2-dihydroquinoline-4-carboxamide (**6a**). To obtain compound **6a** the solid residue was triturated with water (15 mL). Pale brown solid (0.114 g; 92.4 %); m.p.: >300 °C; ^{1}**H NMR** (400 MHz, DMSO-d_6): δ (ppm) 11.98 (s, 1H, Ar-NH-CO), 10.23 (s, 1H, Ar-CO-NH-Ar), 9.40 (t, 1H, *J* = 6.0 Hz, $\underline{NH}$-CH_2), 7.96 (d, 2H, *J* = 8.3 Hz, Ar-H), 7.80–7.76 (m, 2H, Ar-H), 7.72 (dd, 1H, *J* = 8.2, 1.2 Hz, Ar-H), 7.58–7.49 (m, 3H, Ar-H), 7.39–7.32 (m, 3H, Ar-H), 7.21 (ddd, 1H, *J* = 8.2, 7.2, 1.2 Hz, Ar-H), 7.10 (ddd, 1H, *J* = 8.2, 7.2, 1.2 Hz, Ar-H), 6.60 (d, 1H, *J* = 1.7 Hz, Ar-H), 4.58 (d, 2H, *J* = 6.0 Hz, $NH\underline{CH_2}$); ^{13}**C NMR** (101 MHz, DMSO-d_6): δ (ppm) 165.95 (Ar-CO-NH-Ar), 165.36 (Ar-$\underline{CO}$-NH-CH_2), 161.28 (Ar-NH-CO) 145.99, 142.70, 139.29, 139.22, 133.69, 130.94, 128.65, 127.94, 127.15, 125.86, 123.66, 122.14, 120.36, 119.95, 116.16, 115.75, (20 × C-Ar) 42.15 ($NHCH_2$); **HRMS** (ESI+): m/z calcd for $C_{24}H_{20}N_3O_3$ ($[M+H]^+$) 398.1499, found 398.1498; **IR** (ATR): ν (cm^{-1}) 3275, 2989, 2963, 2886, 2854, 1668, 1639, 1601, 1530, 1503, 1435, 1396, 1324, 1295, 1265, 1242,1037, 895, 862, 748, 728, 689, 658, 649, 630, 589, 553, 524, 510.

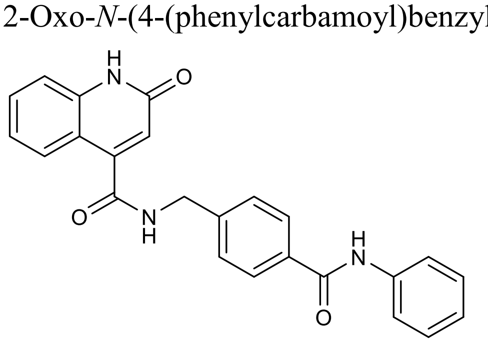

2-Oxo-*N*-(4-(pyridin-4-ylcarbamoyl)benzyl)-1,2-dihydroquinoline-4-carboxamide (**6b**). To obtain

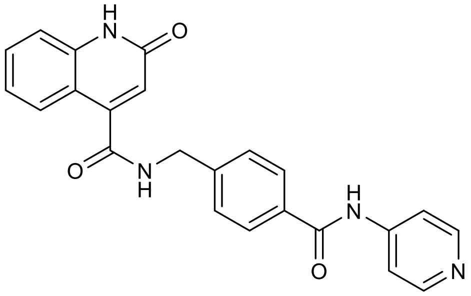

compound **6b** the solid residue was washed with water (2 × 10 mL), Et_2O (10 mL) and MeOH. Light yellow solid (0.032 g; 17.5 %); m.p.: >300 °C; **^{1}H NMR** (400 MHz, DMSO-d_6): δ (ppm) 11.98 (s, 1H, Ar-NH-CO), 10.59 (s, 1H, Ar-CO-NH-Ar), 9.41 (t, 1H, *J* = 6.0 Hz, NH-CH_2), 8.48 (s, 2H, Ar-H), 7.98 (d, 2H, *J* = 8.3 Hz, Ar-H), 7.80 (d, 2H, *J* = 8.3 Hz, Ar-H), 7.71 (d, 1H, *J* = 8.2 Hz, Ar-H), 7.57-7.51 (m, 3H, Ar-H), 7.36 (d, 1H, *J* = 8.2 Hz, Ar-H), 7.20 (ddd, 1H, *J* = 8.2, 7.1, 1.3 Hz, Ar-H), 6.60 (d, 1H, *J* = 1.7 Hz, Ar-H), 4.59 (d, 2H, *J* = 6.0 Hz, NHCH_2); **^{13}C NMR** (101 MHz, DMSO-d_6): δ (ppm) 166.31 (Ar-NH-CO-Ar), 165.97 (Ar-CO-NH-CH_2), 161.28 (Ar-NH-CO), 150.31, 146.03, 145.96, 143.39, 139.29, 132.92, 130.96, 128.17, 127.25, 125.85, 122.15, 119.97, 116.14, 115.76, 114.04 (19 × C-Ar), 42.20 (NHCH_2); **HRMS** (ESI+): m/z calcd for $C_{23}H_{19}N_4O_3$ ($[M+H]^+$) 399.1452, found 399.1450; **IR** (ATR): ν (cm^{-1}) 3222, 3162, 3080, 3031, 2905, 2835, 1649, 1585, 1563, 1542, 1503, 1413, 1399, 1329, 1294, 1256, 1205, 1180, 1158, 989, 962, 889, 828, 799, 779, 756, 734, 697, 579, 536, 510.

Ethyl (1*S*,2*R*)-2-(4-((2-oxo-1,2-dihydroquinoline-4-carboxamido)methyl)benzamildo)cyclohexane-1-carboxylate (**6c**). To obtain compound **6c** the solid residue was washed with water (10 mL), 10 %

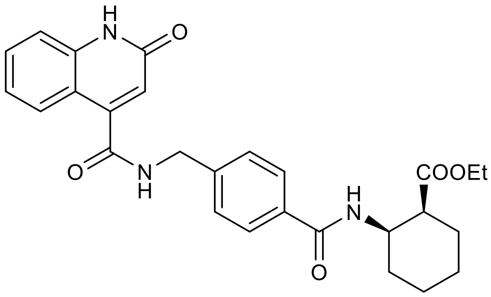

citric acid (3-5 mL) and Et_2O (10 mL). The solid crude product was further purified by silica gel column chromatography using a 9:1 mixture of DCM/MeOH as eluent. White solid (0.047 g; 32.0 %); m.p.: 195.2-196.3 °C; **^{1}H NMR** (400 MHz, DMSO-d_6): δ (ppm) 11.97 (s, 1H, Ar-NH-CO), 9.36 (t, 1H, *J* = 6.0 Hz, NH-CH_2), 8.02 (d, 1H, *J* = 8.4 Hz, NH-cyclohex.), 7.76 (d, 2H, *J* = 8.3 Hz, Ar-H) 7.69 (dd, 1H, *J* = 8.3, 1.3 Hz, Ar-H), 7.54 (ddd, 1H, *J* = 8.3, 7.2, 1.3 Hz, Ar-H), 7.43 (d, 2H, *J* = 8.3 Hz, Ar-H), 7.35 (dd, 1H, *J* = 8.3, 1.3 Hz, Ar-H), 7.19 (ddd, 1H, *J* = 8.3, 7.2, 1.3 Hz, Ar-H), 6.57 (d, 1H, *J* = 2.0 Hz, Ar-H), 4.53 (d, 2H, *J* = 6.0 Hz, CH_2NH), 4.41-4.33 (m, 1H, H-cyclohex.), 4.14–3.86 (m, 2H, CH_2CH_3), 2.94–2.74 (m, 1H, H-cyclohex.), 2.04–1.90 (m, 1H, H-cyclohex.), 1.78 (t, 1H, *J* = 9.9 Hz, H-cyclohex.), 1.75–1.53 (m, 4H, H-cyclohex.), 1.48–1.30 (m, 2H, H-cyclohex.), 1.08 (t, 3H, *J* = 7.1 Hz, CH_2CH_3); **^{13}C NMR** (101 MHz, DMSO-d_6): δ (ppm) 173.02 (-COO-), 166.07(Ar-CO-NH-cyclohex.), 165.88 (Ar-CO-NH-CH_2), 161.25 (Ar-NH-CO), 145.99, 142.05, 139.27, 133.64, 130.92, 127.70, 126.85, 125.83, 122.11, 119.91, 116.13, 115.72 (14 × Ar-C), 59.66 (CH_2CH_3), 47.32, 43.88, (2 × C-cyclohex.) 42.14 (NHCH_2), 32.39, 29.19, 24.25, 22.87 (4 × C-cyclohex.), 14.06 (CH_2CH_3); **HRMS** (ESI+): m/z calcd for $C_{27}H_{30}N_3O_5$ ($[M+H]^+$) 476.2191, found 476.2178; **IR** (ATR): ν (cm^{-1}) 3275, 2932, 2856, 1726, 1669, 1639, 1535, 1502, 1434, 1282, 1256, 1217, 1183, 1158, 1112, 1021, 891, 751, 728, 656, 646, 553, 509.

N-(4-(Morpholine-4-carbonyl)benzyl)-2-oxo-1,2-dihydroquinoline-4-carboxamide (**6d**). To obtain

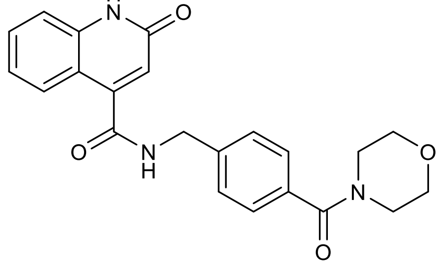

compound **6d** DCM (30 mL) and 10 % citric acid (20 mL) were added in the reaction mixture and the two phases were separated in the separating funnel. The water phase was washed with DCM (20 mL). The combined organic layers were washed with diluted 0.1 M HCl (pH = 4), brine (2 × 20 mL), dried over Na_2SO_4, filtered, concentrated under reduced pressure and recrystallized from EtOH. White solid (0.020 g; 16.5 %); m.p.:

249.2-252.5 °C; **^{1}H NMR** (400 MHz, DMSO-d_6): δ (ppm) 11.97 (s, 1H, Ar-NH-CO), 9.35 (t, 1H, J = 6.0 Hz, NH-CH_2), 7.70 (dd, 1H, J = 8.3, 1.3 Hz, Ar-H), 7.54 (ddd, 1H, J = 8.3, 7.1, 1.3 Hz, Ar-H), 7.46–7.40 (m, 4H, Ar-H), 7.36 (d, 1H, J = 8.3 Hz, Ar-H), 7.19 (ddd, 1H, J = 8.3, 7.1, 1.3 Hz, Ar-H), 6.59 (s, 1H, Ar-H), 4.53 (d, 2H, J = 6.0 Hz, NHCH_2), 3,60 (s, 8H, H-morpholine); **^{13}C NMR** (101 MHz, DMSO-d_6): δ (ppm) 169.00 (Ar-CO-morpholine), 165.92 (Ar-CO-NH-CH_2), 161.27 (Ar-NH-CO), 145.97, 140.54, 139.27, 134.23, 130.93, 127.36, 127.22, 125.87, 122.13, 119.93, 116.14, 115.73 (14 × Ar-C), 66.11 (2 × C-morpholine), 47.83 (2 × C-morpholine), 42.15 (NHCH_2); **HRMS** (ESI+): m/s calcd for $C_{22}H_{22}N_3O_4$ ([M+H]$^+$) 392.1605, found 392.1600; **MS** (ESI+): m/z = 413,90 ([M + Na]$^+$, 100 %), **MS** (ESI-): m/z = 391,15 ([M-H]$^-$, 100 %); **IR** (ATR): ν (cm^{-1}) 3286, 2959, 2850, 2734, 1668, 1637, 1547, 1428, 1396, 1298, 1280, 1256, 1110, 1015, 889, 850, 820, 795, 751, 719, 686, 551, 509.

N-(4-(3-Acetamidopiperidine-1-carbonyl)benzyl)-2-oxo-1,2-dihydroquinoline-4-carboxamide (**6e**).

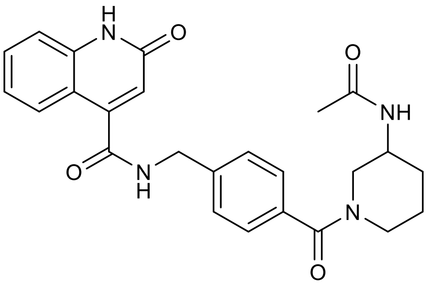

To obtain compound **6e** solid residue was washed with water (20 mL), filtered and air-dried. Light pink solid (0.090 g; 65.2%); m.p.: 248.1-250.3 °C; **^{1}H NMR** (400 MHz, DMSO-d_6): δ (ppm) 11.96 (s, 1H, Ar-NH-CO), 9.34 (t, 1H, J = 6.0 Hz, NH-CH_2), 7.88 (s, 1H, NH-pip.) 7.70 (dd, 1H, J = 8.3, 1.4 Hz, Ar-H), 7.54 (ddd, 1H, J = 8.3, 7.2, 1.4 Hz, Ar-H), 7.44–7.31 (m, 5H, Ar-H), 7.20 (ddd, 1H, J = 8.3, 7.2, 1.4 Hz, Ar-H), 6.58 (d, 1H, J = 1.8 Hz, Ar-H), 4.53 (d, 2H, J = 6.0 Hz, CH_2NH), 4.07 (m, 1H, H-pip.) 3.67 (s,1H, H-pip.), 3.45–3.38 (m, 1H, H-pip.), 3.21 (s, 1H, H-pip.), 3.08 – 2.91 (m, 1H, H-pip.) , 1.82 (s, 3H, $COCH_3$), 1.70 (m, 2H, H-pip.), 1.45 (s, 2H, H-pip.); **^{13}C NMR** (101 MHz, DMSO-d_6): δ (ppm) 169.15 (Ar-CO-pip.), 168.75 (CH_3-CO-NH-pip.), 165.92 (Ar-CO-NH-CH_2), 161.28 (Ar-NH-CO), 146.02, 140.21, 139.27, 134.90, 130.92, 127.09, 125.88, 122.14, 119.89, 116.16, 115.72 (14 × C-Ar), 51.41, 47.21, 45.27 (3 × C-pip.), 42.16 (NHCH_2), 30.07, 29.50 (2 × C-pip.), 22.61 (CH_3CO); **HRMS** (ESI+): m/z calcd for $C_{25}H_{27}N_4O_4$ ([M+H]$^+$) 447.2027, found 447.2022; **MS** (ESI+): m/z = 469.00 ([M+Na]$^+$, 100 %), **MS** (ESI-): m/z = 445.00 ([M-H]$^-$, 100 %). **IR** (ATR): ν (cm^{-1}) 3274, 2994, 2935, 2854, 1637, 1605, 1543, 1468, 1432, 1396, 1368, 1297, 1263, 1036, 980, 893, 861, 751, 724, 691, 655, 639, 599, 552, 525, 508.

Ethyl 1-(4-((2-oxo-1,2-dihydroquinoline-4-carboxamido)methyl)benzamido)cyclohexane-1-carboxylate (**6f**). To obtain compound **6f** solid residue was washed with water (10 mL), filtered and air-dried.

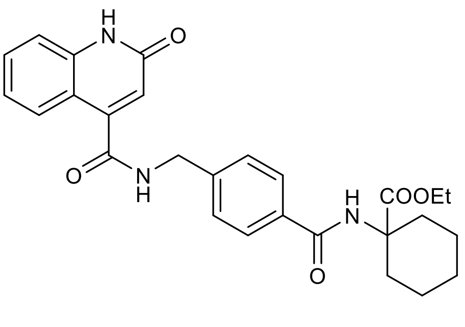

Brown solid (0.122 g, 83.0%); m.p.: 156.4-161.2 °C; **^{1}H NMR** (400 MHz, DMSO-d_6): δ (ppm) 11.97 (s, 1H, Ar-NH-CO), 9.38 (t, 1H, J = 5.7 Hz, NH-CH_2), 8.34 (s, 1H, CO-NH-cyclohex.), 7.81 (d, 2H, J = 8.0 Hz, Ar-H), 7.69 (d, 1H, J = 8.0 Hz, Ar-H), 7.54 (ddd, 1H, J = 8.0, 7.1, 0.4 Hz, Ar-H), 7.49–7.39 (m, 3H, Ar-H), 7.20 (ddd, 1H, J = 8.0, 7.1, 0.4 Hz, Ar-H), 6.58 (s, 1H, Ar-H) 4.54 (d, 2H, J = 5.7 Hz, NHCH_2), 4.04 (q, 2H, J = 7.0 Hz, CH_2CH_3), 2.08 (d, 2H, J = 13.3 Hz, H-cyclohex.), 1.76 (t, 2H, J = 11.0 Hz, H-cyclohex.), 1.54 (s, 5H, H-cyclohex.), 1.25 (d, 1H, J = 6.3 Hz, H-cyclohex.), 1.12 (t, 3H, J = 7.0 Hz, CH_2CH_3); **^{13}C NMR** (101 MHz, DMSO-d_6): δ (ppm) 174.19 (-COO-), 166.83 (CO-NH-cyclohex.), 166.17 (Ar-CO-NH-CH_2), 161.60 (Ar-NH-CO), 146.30, 142.52, 139.37, 133.44, 131.24, 128.07, 127.15, 126.01, 122.48, 119.92, 116.33, 115.99, (14 × Ar-C) 60.29 (C-cyclohex.), 58.74 (CH_2CH_3), 42.39 (NHCH_2), 32.05 (2 × C-cyclohex.), 25.20 (C-cyclohex.), 21.49 (2 × C-cyclohex.), 14.32 (CH_2CH_3); **HRMS**

(ESI+): m/s calcd for $C_{27}H_{30}N_3O_5$ ($[M+H]^+$) 476.2180, found 476.2179; **IR** (KBr): ν (cm^{-1}) 2931, 2859, 2360, 2340, 1733, 1671, 1640, 1531, 1501, 1435, 1396, 1326, 1290, 1236, 1203, 1163, 1066, 751, 555, 511.

General procedure for the synthesis of 7.

Amine (1 eq) was dissolved in ice-cold anhydrous DMF under an argon atmosphere. Et_3N (5 eq) and compound **F20** (1 eq) were added and stirred for 10 minutes. After 10 minutes HOBt (1.2 eq) and EDC (1.3 eq) were added. The reaction mixture was stirred overnight at room temperature under an argon atmosphere. The solution was concentrated under reduced pressure and various ways of purification were used depending on the properties of individual compound. Solid residue of 7 was triturated with DCM to obtain compounds **7a, 7c** and **7d.** Compound **7b** was additionally washed with Et_2O and recrystallized from EtOH.

N-(4-(((1*R*,4*R*)-4-Hydroxycyclohexyl)carbamoyl)benzyl)-2-oxo-1,2-dihydroquinoline-4-carboxamide (**7a**). White-yellow crystals (0.116 g; 89.2 %); m.p.: 277 °C; **^{1}H NMR** (400 MHz,

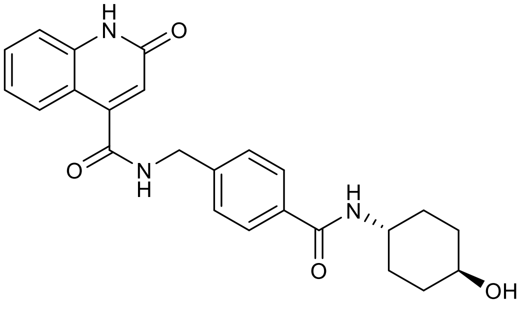

DMSO-d_6) δ (ppm) 11.97 (s, 1H, Ar-<u>NH</u>-CO), 9.37 (t,1H, *J* = 5.9 Hz, <u>NH</u>-CH_2), 8.16 (d, 1H, *J* = 7.9 Hz, CO-<u>NH</u>-cyclohex.), 7.82 (d, 2H, *J* = 8.2 Hz, Ar-H), 7.68 (d, 1H, *J* = 8.1 Hz, Ar-H), 7.57 – 7.50 (m, 1H, Ar-H), 7.41 (d, 2H, *J* = 8.2 Hz, Ar-H), 7.36 (d, 1H, *J* = 8.2 Hz, Ar-H), 7.22 – 7.15 (m, 1H, Ar-H), 6.56 (d, 1H, *J* = 1.2 Hz, Ar-H), 4.57 (d, 1H, *J* = 3.7 Hz, OH), 4.52 (d, 1H, *J* = 5.9 Hz, H-cyclohex.), 3.75 – 3.65 (m, 1H, H-cyclohex.), 1.81 (t, 4H, *J* = 14.7 Hz, H-cyclohex.), 1.45 – 1.12 (m, 4H, H-cyclohex.); **^{13}C NMR** (101 MHz, DMSO-d_6): δ (ppm): 166.33 (<u>CO</u>-NH-cyclohex.), 165.78 (Ar-<u>CO</u>-NH-CH_2), 161.69 (Ar-NH-CO), 146.43, 142.43, 139.72, 133.98, 131.32, 127.93, 127.36, 126.25, 122.54, 120.33, 116.58, 116.19 (14 × Ar-C), 68.80 (C-cyclohex.), 48.36 (C-cyclohex.), 42.59 (<u>CH_2</u>-NH), 34.71 (2 × C-cyclohex.), 30.78 (2 × C-cyclohex.); **HRMS** (ESI+): m/z calcd for $C_{24}H_{26}N_3O_4$ ($[M + H]^+$) 420.19178, found 420,19101; **MS** (ESI-): m/z = 419.20 ($[M-H]^-$, 100 %); **IR** (ATR): ν (cm^{-1}) 3282, 2933, 2857, 1662, 1642, 1536, 1504, 1476, 1440, 1394, 1333, 1289, 1235, 1189, 1163, 1084, 1052, 983, 946, 896, 874, 842, 801, 778, 752, 722, 683, 651, 617, 555, 524, 513.

Ethyl (1*R*,4*R*)-4-((4-((2-oxo-1,2-dihydroquinoline-4 carboxamido)methyl)benzamido)methyl)cyclohexane-1-carboxylate (**7b**). Dark yellow crystals

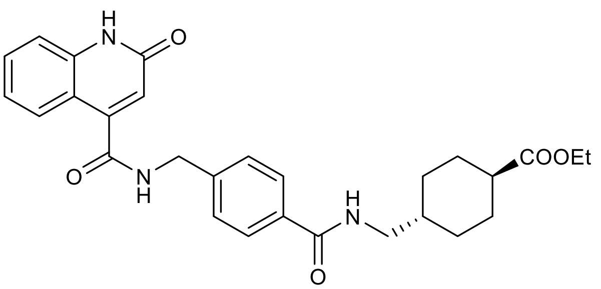

(0.128 g; 86.8 %); m.p.: 263 °C; **^{1}H NMR** (400 MHz, DMSO-d_6): δ (ppm) 11.98 (s, 1H, Ar-NH-CO), 9.38 (t, 1H, *J* = 5.8 Hz, <u>NH</u>-CH_2-Ar), 8.46 (t, 1H, *J* = 5.6 Hz, <u>NH</u>-CH_2-cyclohex.), 7.84 (d, 2H, *J* = 8.1 Hz, Ar-H), 7.69 (d, 1H, *J* = 8.1 Hz, Ar-H), 7.54 (t, 1H, *J* = 7.7 Hz, Ar-H), 7.43 (d, 2H, *J* = 8.0 Hz, Ar-H), 7.37 (d, 1H, *J* = 8.2 Hz, Ar-H), 7.19 (t, 1H, *J* = 7.6 Hz, Ar-H), 6.57 (s, 1H, Ar-H), 4.53 (d, 2H, *J* = 5.8 Hz, NH-<u>CH_2</u>-Ar), 4.03 (q, 2H, *J* = 7.1 Hz, CH_3<u>CH_2</u>), 3.11 (t, 2H, *J* = 6.2 Hz, NH-<u>CH_2</u>-cyclohex.), 2.22 (t, 1H, *J* = 12.1 Hz, H-cyclohex.), 1.89 (d, 2H, *J* = 11.5 Hz, H-cyclohex.), 1.77 (d, 2H, *J* = 13.3 Hz, H-cyclohex.), 1.51 (s, 1H, H-cyclohex.), 1.36 – 1.24 (m, 2H, H-cyclohex.), 1.16 (t, 3H, *J* = 7.1 Hz, <u>CH_3</u>CH_2), 1.04 – 0.91 (m, 2H, H-cyclohex.). *Alkaline hydrolysis of **7b** was performed without detailed intermediate compound characterization.

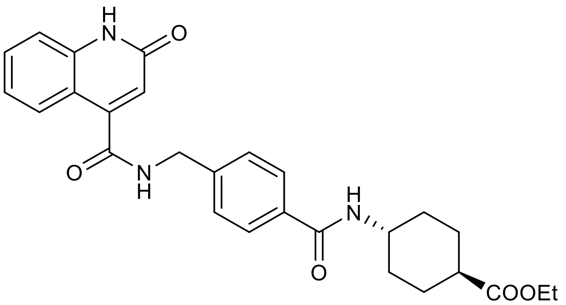

Ethyl (1*R*,4*R*)-4-(4-((2-oxo-1,2-dihydroquinoline-4-carboxamido)methyl) benzamido)cyclohexane-1-carboxylate (**7c**). Pale grey crystals (0.115 g; 77.9 %)

*Alkaline hydrolysis of **7c** was performed without intermediate compound characterization.

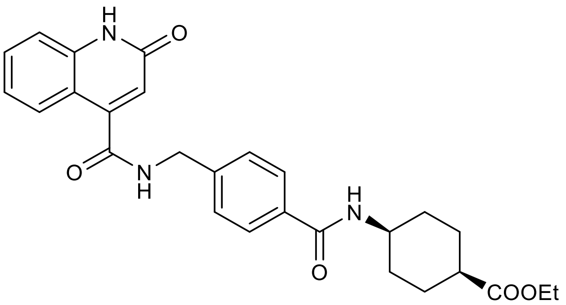

Ethyl (1*S*,4*S*)-4-(4-((2-oxo-1,2-dihydroquinoline-4-carboxamido)methyl) benzamido)cyclohexane-1-carboxylate (**7d**). Pale grey crystals (0.131 g; 89.12 %)

*Alkaline hydrolysis of **7d** was performed without intermediate compound characterization.

General procedure for the synthesis of compounds 8.

Compound **6f** or **7b-d** (1 eq) was dissolved in 5 mL of EtOH and 1 M NaOH (5 eq) was added. The reaction mixture was stirred for 72 h at room temperature. 10 mL of water was added in reaction mixture and EtOH was evaporated under reduced pressure. The water phase was acidified (pH 1-2) with 1 M HCl and precipitate was filtered and dried to obtain compound **8**.

1-(4-((2-Oxo-1,2-dihydroquinoline-4-carboxamido)methyl)benzamido)cyclohexane-1-carboxylic acid (**8a**). White solid (0.039 g; 13.6 %); m.p.: 243.5-246.8 °C; **^{1}H NMR** (400 MHz, DMSO-d_6): δ (ppm) 12.14 (s, 1H, COOH), 11.97 (s, 1H, Ar-NH-CO), 9.38 (t, 1H, J = 6.0 Hz, NH-CH$_2$), 8.19 (s, 1H, CO-NH-cyclohex.), 7.84 (d, 2H, J = 8.3 Hz, Ar-H), 7.70 (dd, 1H, J = 8.3, 1.3 Hz, Ar-H), 7.54 (ddd, 1H, J = 8.3, 7.2, 1.3 Hz, Ar-H), 7.44 (d, 2H, J = 8.3 Hz, Ar-H), 7.36 (dd, 1H, J_1 = 8.3, 1.3 Hz, Ar-H), 7.20 (ddd, 1H, J = 8.3, 7.2, 1.3 Hz, Ar-H), 6.58 (s, 1H, Ar-H), 4.54 (d, 2H, J = 6.0 Hz, NHCH$_2$), 2.12 (d, 2H, J = 13.3 Hz, H-cyclohex.), 1.74 (dt, 2H, J = 14.0, 7.4, H-cyclohex.), 1.54 (s, 5H, H-cyclohex.), 1.29 (d, 1H, J = 8.3 Hz, H-cyclohex.); **^{13}C NMR** (101 MHz, DMSO-d_6): δ (ppm) 175.72 (COOH), 166.34 (CO-NH-cyclohex.), 165.89 (Ar-CO-NH-CH$_2$), 161.26 (Ar-NH-CO), 146.01, 142.24, 139.27, 133.45, 130.93, 127.91, 126.85, 125.84, 122.14, 119.91, 116.14, 115.73 (14 × Ar-C), 58.34 (C-cyclohex.), 42.18 (NHCH$_2$), 31.78, 25.16, 21.36 (5 × C-cyclohex.); **HRMS** (ESI-): m/z calcd for $C_{25}H_{24}N_3O_5$ ([M-H]$^-$) 446.1721, found 446.1726; **IR** (ATR): ν (cm^{-1}) 3242, 3063, 2929, 2863, 1698, 1669, 1638, 1534, 1503, 1327, 1284, 1263, 1240, 1190, 1169, 990, 947, 879, 771, 750, 682, 659, 628, 559, 514.

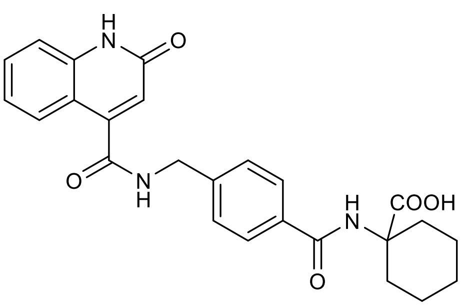

(1*R*,4*R*)-4-((4-((2-Oxo-1,2-dihydroquinoline-4-carboxamido)methyl)benzamido) methyl)cyclohexane-1-carboxylic acid (**8b**). Dark yellow crystals (0.082 g; 73.9%), m.p.: 263 °C; ^{1}**H NMR** (400 MHz, DMSO-d_6) δ (ppm) 11.97 (s, 2H, COOH and Ar-CONH-Ar), 9.36 (t, 1H, *J* = 5.9 Hz, NH-CH_2-Ar), 8.43 (t, 1H, *J* = 5.5 Hz, NH-CH_2-cyclohex.), 7.84 (d, 2H, *J* = 8.2 Hz, Ar-H), 7.70 (d, 1H, *J* = 8.0 Hz, Ar-H), 7.54 (t, 1H, *J* = 7.7 Hz, Ar-H), 7.43 (d, 2H, *J* = 8.2 Hz, Ar-H), 7.36 (d, 1H, *J* = 8.2 Hz, Ar-H), 7.20 (t, 1H, *J* = 7.6 Hz, Ar-H), 6.58 (s, 1H, Ar-H), 4.54 (d, 2H, *J* = 5.7 Hz, NH-CH_2-Ar), 3.11 (t, 2H, *J* = 6.0 Hz, NH-CH_2-cyclohex.), 2.12 (d, 1H, *J* = 11.8 Hz, H-cyclohex.), 1.90 (d, 2H, *J* = 12.1 Hz, H-cyclohex.), 1.77 (d, 2H, *J* = 11.8 Hz, H-cyclohex.), 1.50 (s, 1H, H-cyclohex.), 1.26 (dd, 2H, *J* = 24.6, 11.1 Hz, H-cyclohex.), 0.96 (dd, 2H, *J* = 24.6, 11.1 Hz, H-cxclohex.); ^{13}**C NMR** (101 MHz, DMSO-d_6): δ (ppm) 177.22 (-COOH), 166.50 (CO-NH-CH_2-cyclohex.), 166.34 (Ar-CO-NH-CH_2), 161.70 (Ar-NH-CO), 146.43, 142.46, 139.72, 133.90, 131.34, 130.00, 127.86, 127.75, 127.45, 126.27, 122.55, 120.34, 116.59, 116.18 (14 × Ar-C), 45.70 (CO-NH-CH_2), 43.00 (C-cyclohex.), 42.61 (CO-NH-CH_2), 37.50 (C-cyclohex.), 30.05 (2 × C-cyclohex.), 28.80 (2 × C-cyclohex.); **HRMS** (ESI+): m/z calcd for $C_{26}H_{28}N_3O_5$ ($[M+H]^+$) 462.2023, found 462.2016; **MS** (ESI-): m/z = 462.2 ($[M-H]^-$, 100 %); **IR** (ATR): ν (cm^{-1}) = 3288, 2923, 2036, 1628, 1535, 1504, 1470, 1427, 1394, 1350, 1290, 1263, 1206, 1156, 1135, 1019, 986, 881, 799, 776, 752, 684, 652, 625, 554.

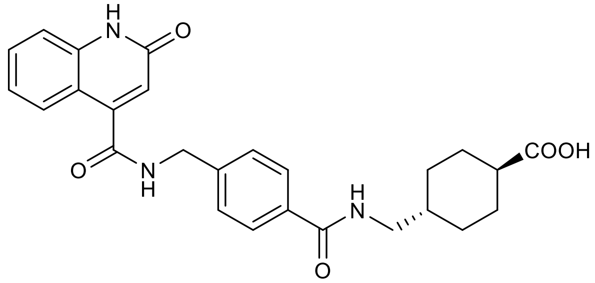

(1*R*,4*R*)-4-(4-((2-Oxo-1,2-dihydroquinoline-4-carboxamido)methyl)benzamido) cyclohexane-1-carboxylic acid (**8c**). Brown crystals (0.037 g; 35.9%); m.p.: 171 °C; ^{1}**H NMR** (400 MHz, DMSO-d_6) δ (ppm) 11.97 (s, 1H, Ar-NHCO-Ar), 9.36 (t, 1H, *J* = 6.1 Hz, CO-NH-CH_2), 8.22 (d, 1H, *J* = 7.6 Hz, CO-NH-cyclohex.), 7.83 (d, 2H, *J* = 7.8 Hz, Ar-H), 7.69 (d, 1H, *J* = 7.9 Hz, Ar-H), 7.54 (t, 1H, *J* = 7.6 Hz, Ar-H), 7.43 (d, 2H, *J* = 7.8 Hz, Ar-H), 7.36 (d, 1H, *J* = 8.3 Hz, Ar-H), 7.20 (t, 1H, *J* = 7.7 Hz, Ar-H), 6.57 (s, 1H, Ar-H), 4.53 (d, 2H, *J* = 5.7 Hz, CO-NH-CH_2), 3.72 (s, 1H, H-cyclohex.), 2.15 (t, 1H, *J* = 9.2 Hz, H-cyclohex.), 1.91 (dd, 3H, *J* = 28.4, 11.4 Hz, H-cyclohex.), 1.76 (s, 1H, H-cyclohex.), 1.51 – 1.27 (m, 4H, H-cyclohex.); ^{13}**C NMR** (101 MHz, DMSO-d_6): δ (ppm) 176.92 (-COOH), 166.33 (CO-NH-CH_2-cyclohex-), 165.71 (Ar-CO-NH-CH_2), 161.69 (Ar-NH-CO), 146.43, 142.45, 139.72, 133.97, 131.34, 127.94, 127.37, 126.26, 122.54, 120.33, 116.58, 116.17 (14 × C-Ar), 48.32 (1 × C-cyclohex.), 42.60 (CO-NH-CH_2), 42.26 (1 × C-cyclohex.), 31.77 (2 × C-cyclohex.), 28.28 (2 × C-cyclohex.); **HRMS** (ESI+): m/z calcd for $C_{25}H_{26}N_3O_5$ ($[M+H]^+$) 448.1863, found 448.1867; **MS** (ESI-): m/z = 447.2 ($[M-H]^-$, 100 %); **IR** (ATR): ν (cm^{-1}) 3278, 2932, 2858, 2111, 2018, 1662, 1641, 1535, 1504, 1427, 1395, 1333, 1287, 1205, 1150, 1034, 944, 903, 875, 778, 753, 723, 684, 651, 555, 505.

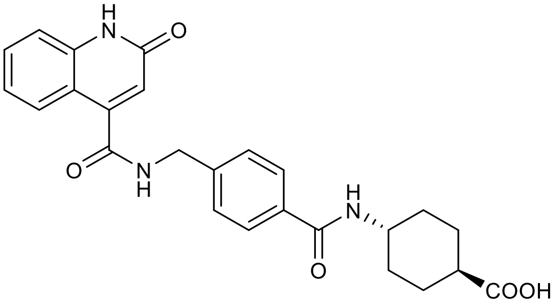

(1*S*,4*S*)-4-(4-((2-Oxo-1,2-dihydroquinoline-4-carboxamido)methyl)benzamido)cyclohexane-1-carboxylic acid (**8d**). Brown crystals (0.038 g; 35.9%); m.p.: 189 °C; **^{1}H NMR** (400 MHz, DMSO-d_6) δ (ppm): 12.16 (s, 1H, COOH), 11.96 (s, 1H, Ar–NHCO-Ar), 9.35 (t, 1H, *J* = 5.6 Hz, NH-CH_2), 8.18 (d, 1H, *J* = 7.2 Hz, CONH-cyclohex.), 7.84 (d, 2H, *J* = 7.9 Hz, Ar-H), 7.69 (d, 1H, *J* = 8.0 Hz, Ar-H), 7.54 (t, 1H, *J* = 7.8 Hz, Ar-H), 7.38 (dd, 3H, *J* = 24.4, 8.0 Hz, Ar-H), 7.19 (t, 1H, *J* = 7.6 Hz, Ar-H), 6.57 (s, 1H, H-Ar), 4.53 (d, 2H, *J* = 5.8 Hz, NH-CH_2), 3.85 (s, 1H, H-cyclohex.), 2.00 (s, 2H, H-cyclohex.), 1.77 – 1.46 (m, 6H, H-cyclohex.); **^{13}C NMR** (101 MHz, DMSO-d_6): δ (ppm): 176.42 (-COOH), 166.33 (CO-NH-cyclohex.), 165.85 (Ar-CO-NH-CH_2), 161.70 (Ar-NH-CO), 146.45, 142.39, 139.72, 134.01, 131.34, 128.04, 127.31, 126.26, 122.55, 120.32, 116.59, 116.17 (14 × C-Ar), 47.50 (1 × C-cyclohex.), 42.61 (CO-NH-CH_2), 39.11 (1 × C-cyclohex.), 29.41 (2 × C-cyclohex.), 25.81 (2 × C-cyclohex.); **HRMS** (ESI+): m/z calcd for $C_{25}H_{25}N_3O_5$ ($[M+H]^+$) 448.1867, found 448.1857; **MS** (ESI-): m/z = 448.1 ($[M-H]^-$, 100 %); **IR** (ATR): ν (cm^{-1}) 3258, 2935, 2007, 1953, 1705, 1637, 1534, 1502, 1431, 1266, 1184, 1134, 1035, 884, 801, 752, 687, 657, 622, 556, 507. *One H-cyclohex. signal is overlapping with the signal for solvent.

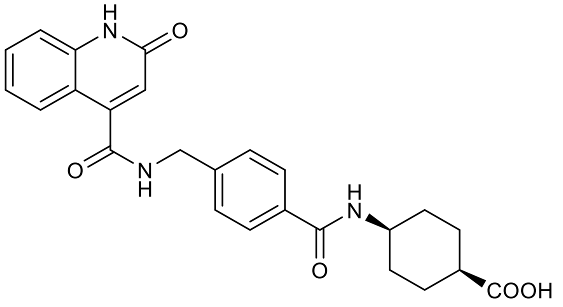

^{1}H and ^{13}C NMR spectra for fragments and compounds

Fragment **1a**: ^{1}H NMR (400 MHz, DMSO-d_6)

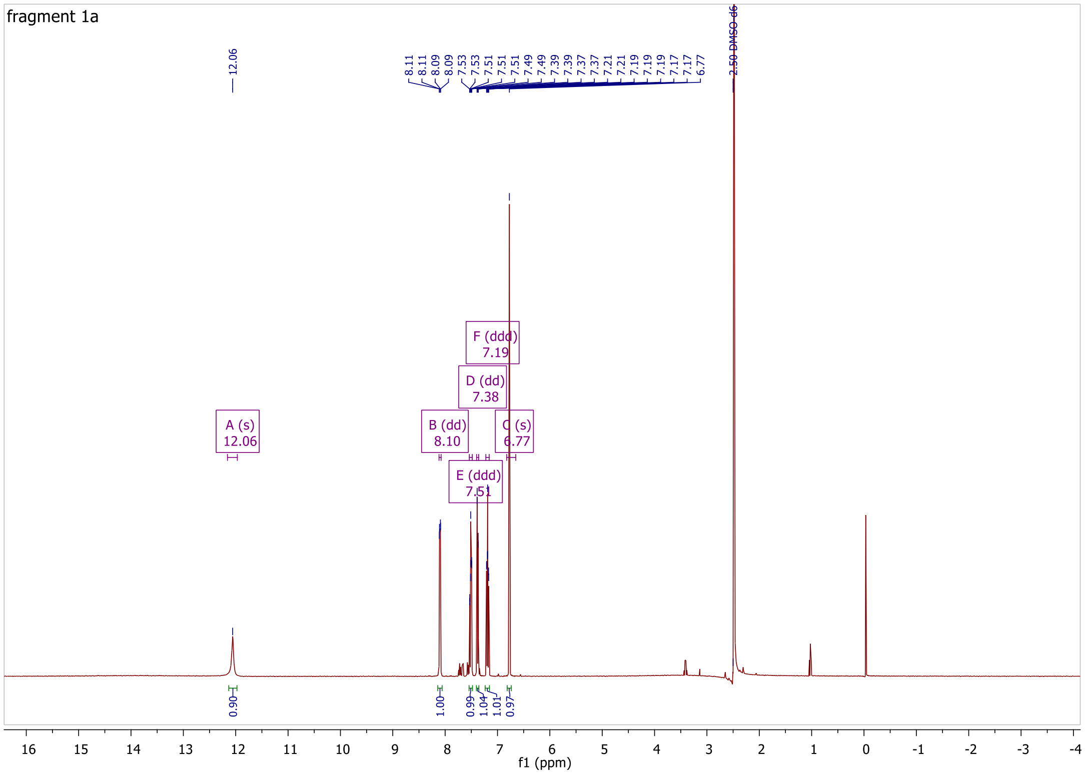

Fragment **1b**: ^{1}H NMR (400 MHz, DMSO-d_6)

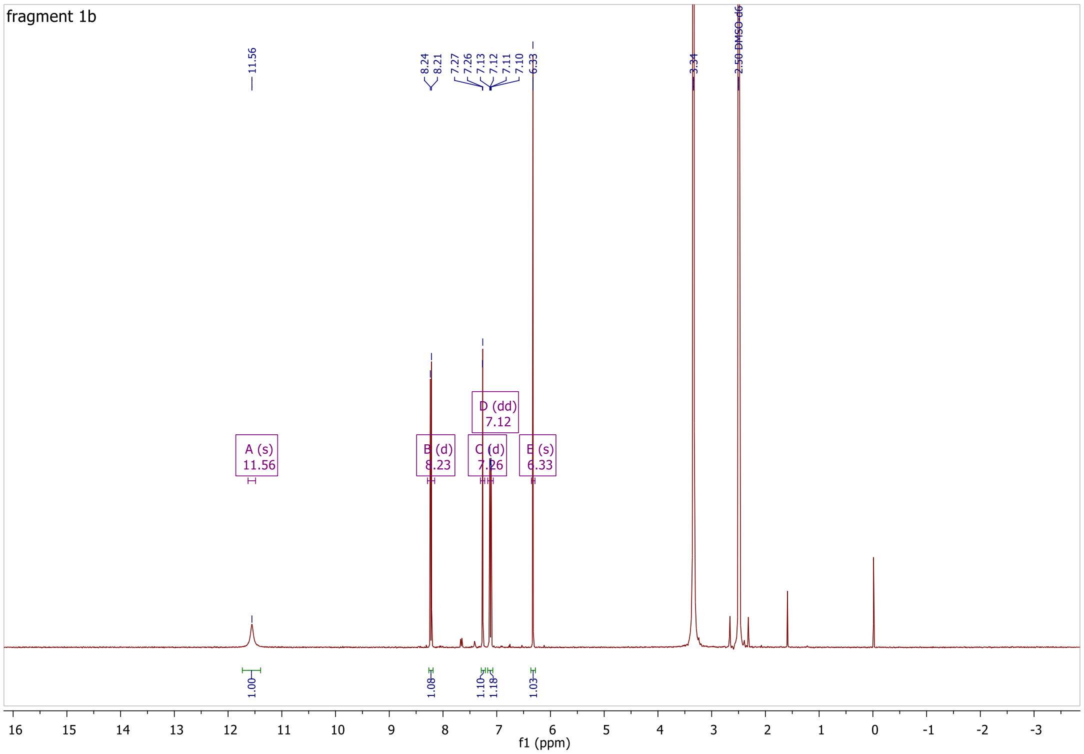

Fragment **2**: ^{1}H NMR (400 MHz, DMSO-d_6)

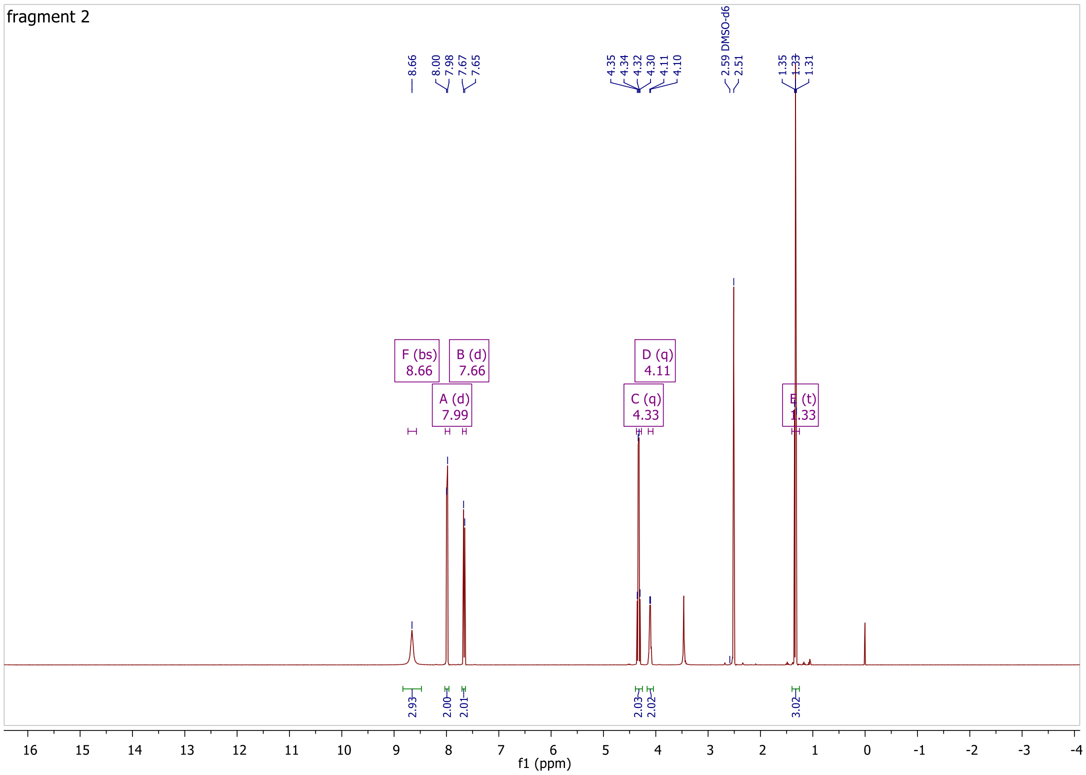

Fragment **F19**: ^{1}H NMR (400 MHz, DMSO-d_6)

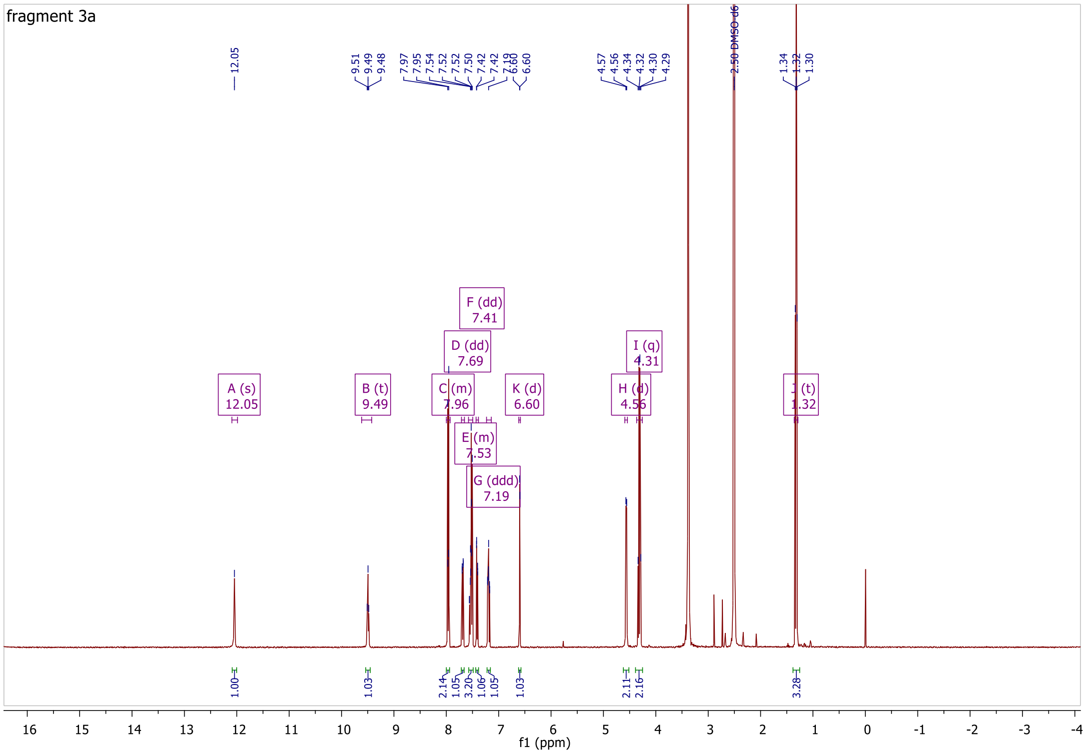

Fragment **F19**: ^{13}C NMR (101 MHz, DMSO-d_6)

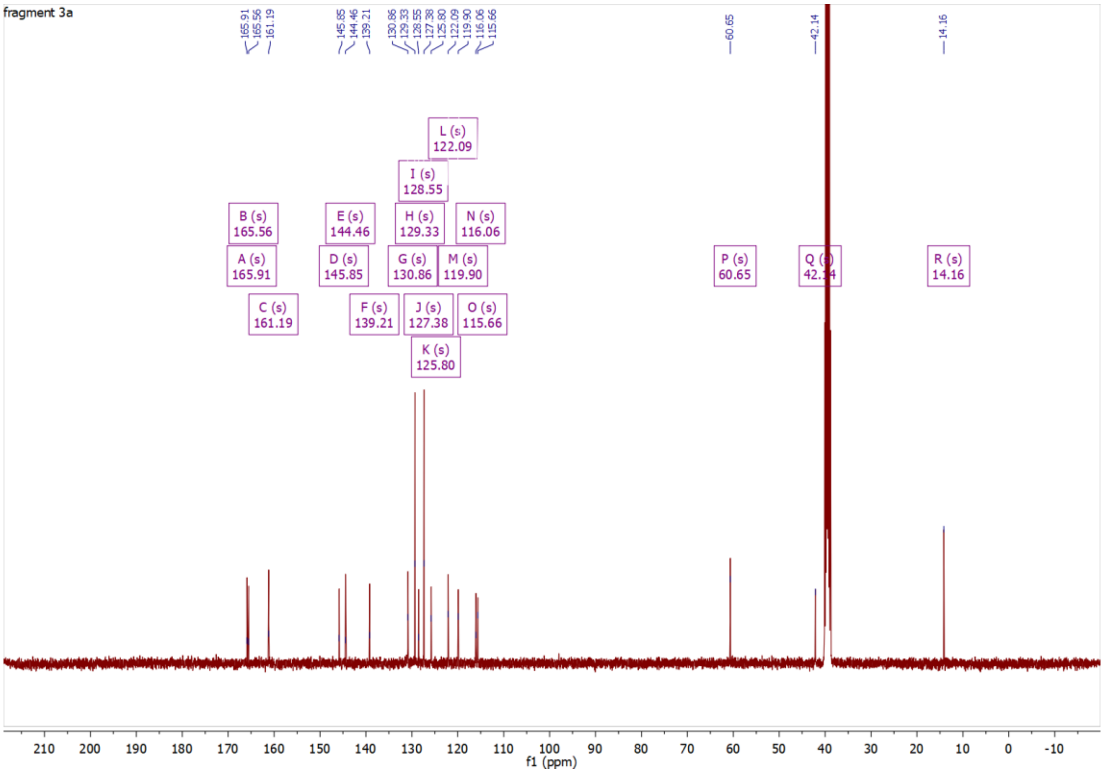

Fragment **3b**: ^{1}H NMR (400 MHz, DMSO-d_6)

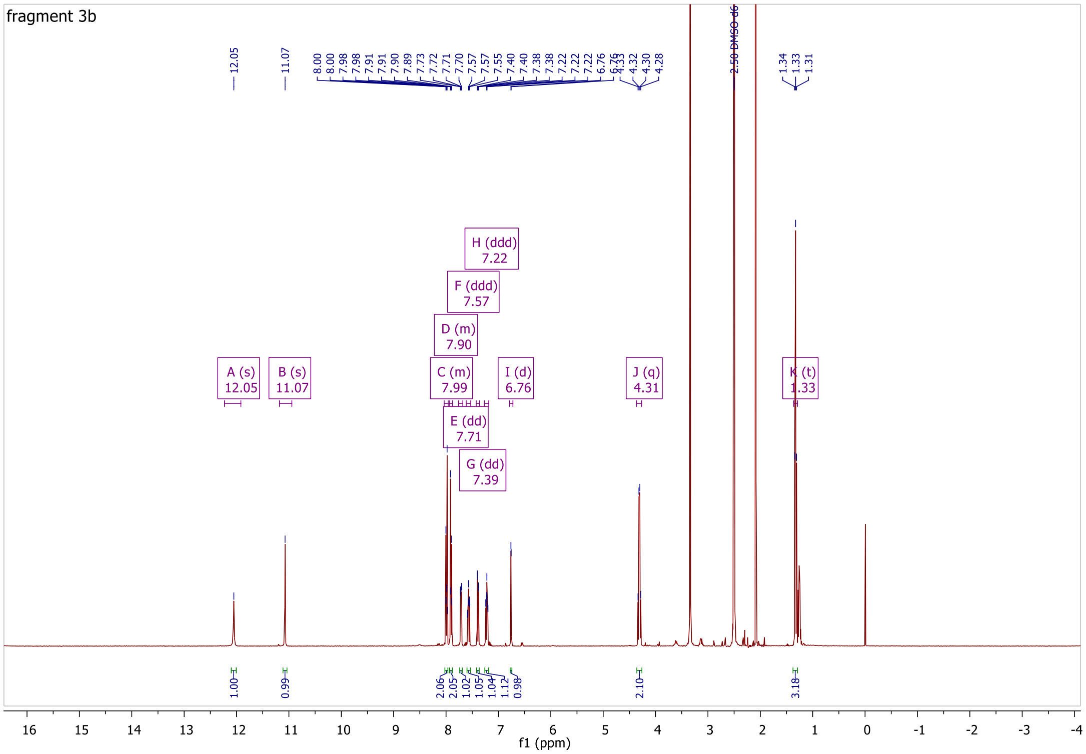

Fragment **3b**: ^{13}C NMR (101 MHz, DMSO-d_6)

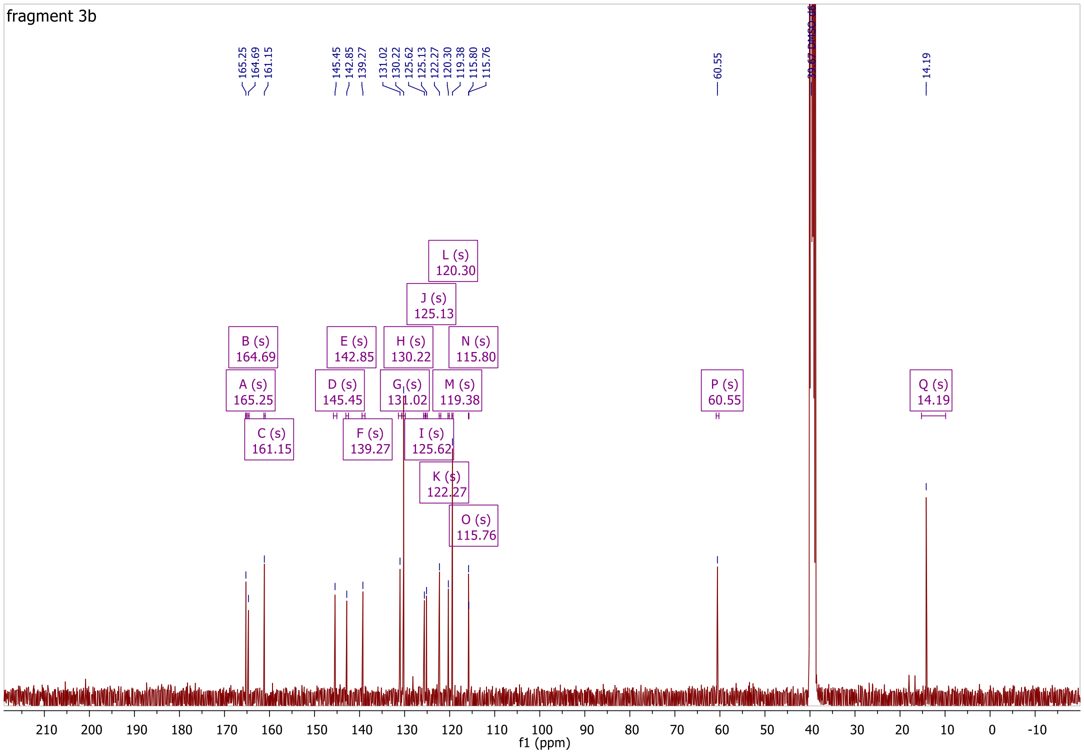

Fragment **3c**: ^{1}H NMR (400 MHz, DMSO-d_6)

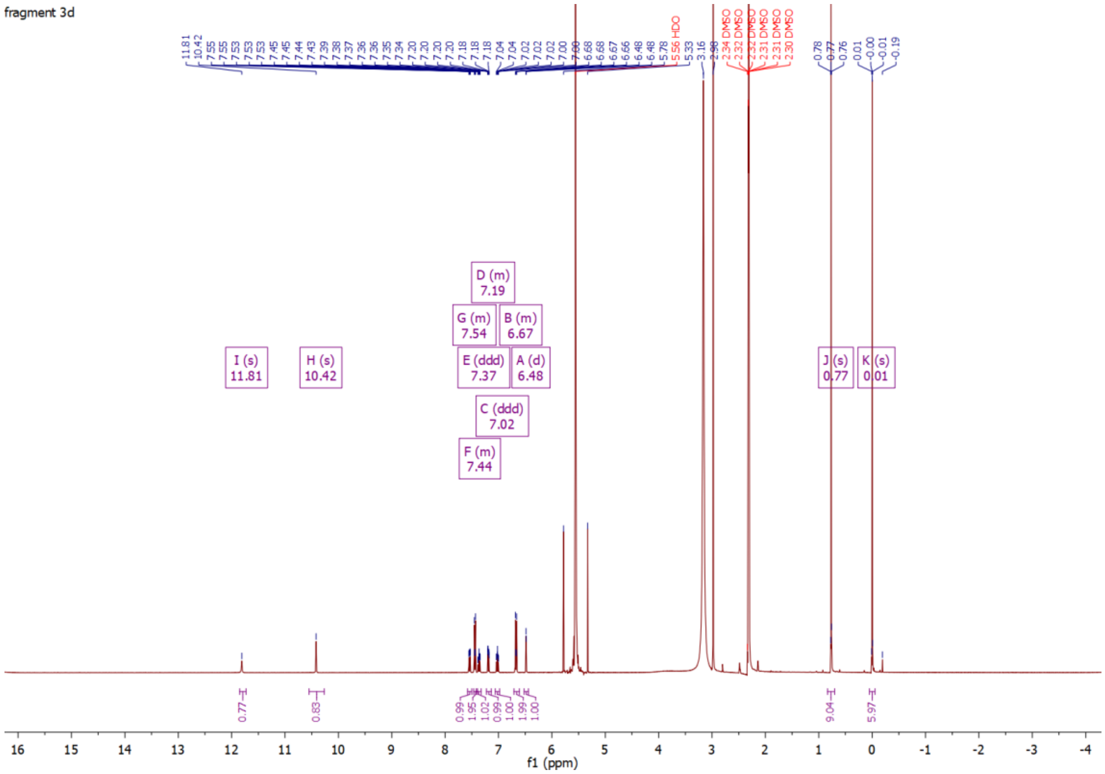

Fragment **F20**: ^{1}H NMR (400 MHz, DMSO-d_6)

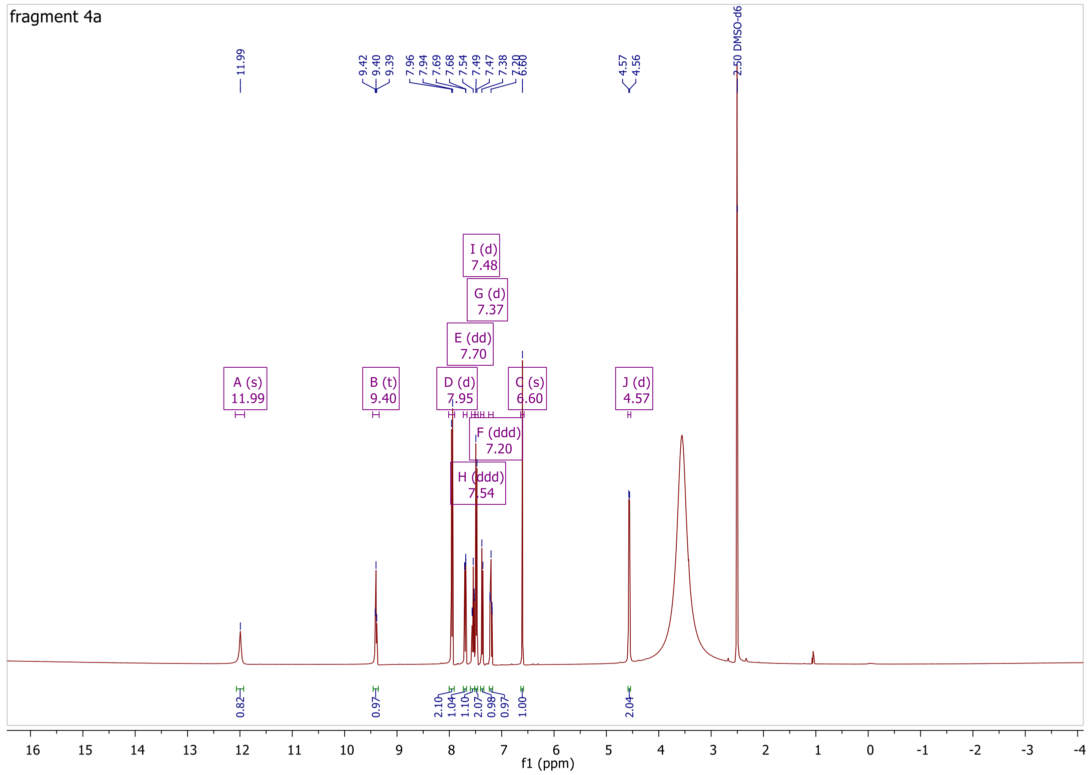

Fragment **F20**: ^{13}C NMR (101 MHz, DMSO-d_6)

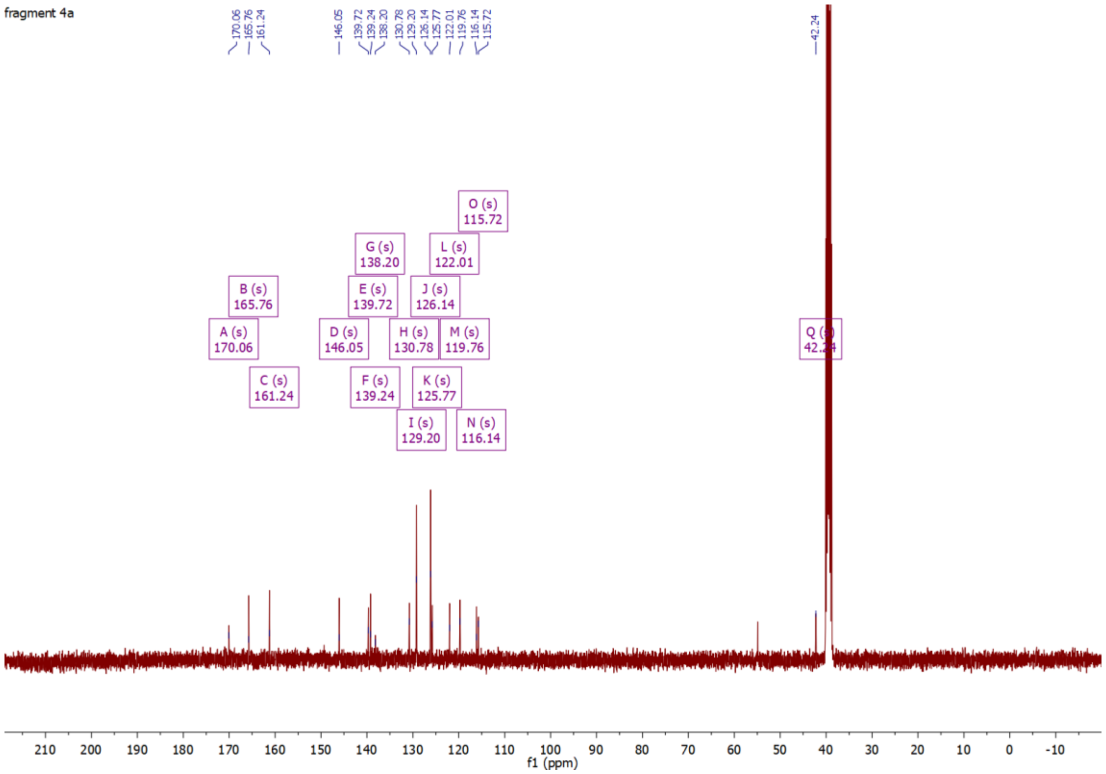

Fragment **4b**: ^{1}H NMR (400 MHz, DMSO-d_6)

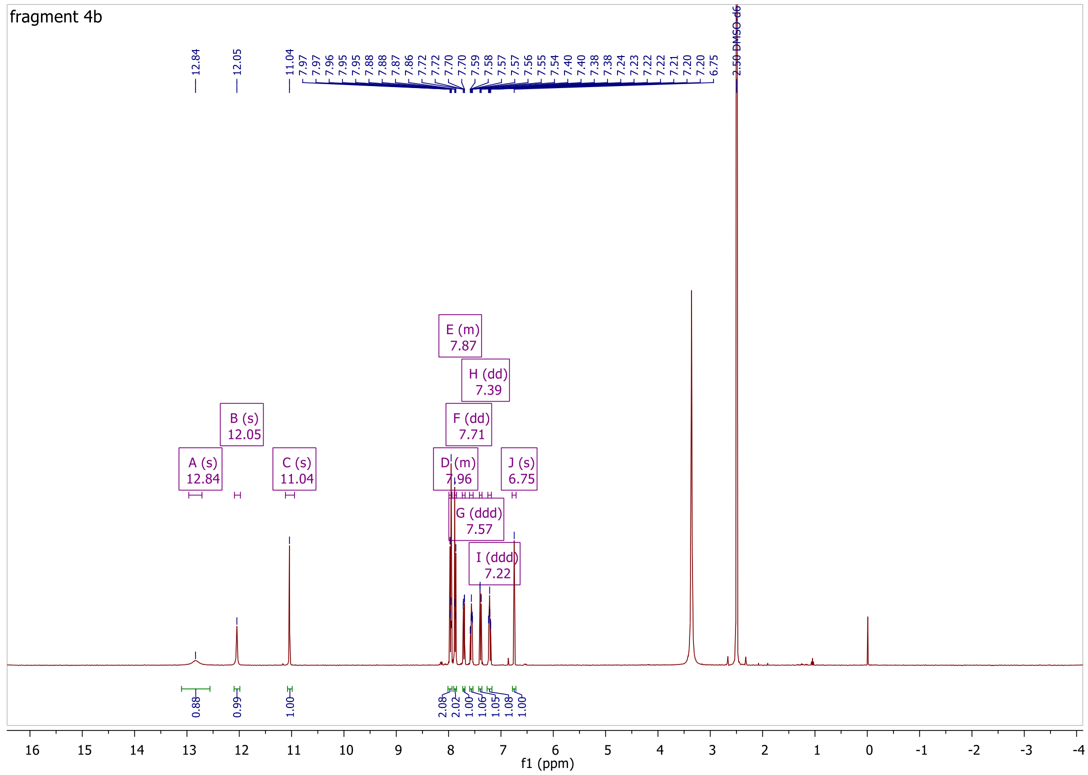

Fragment **4b**: ^{13}C NMR (101 MHz, DMSO-d_6)

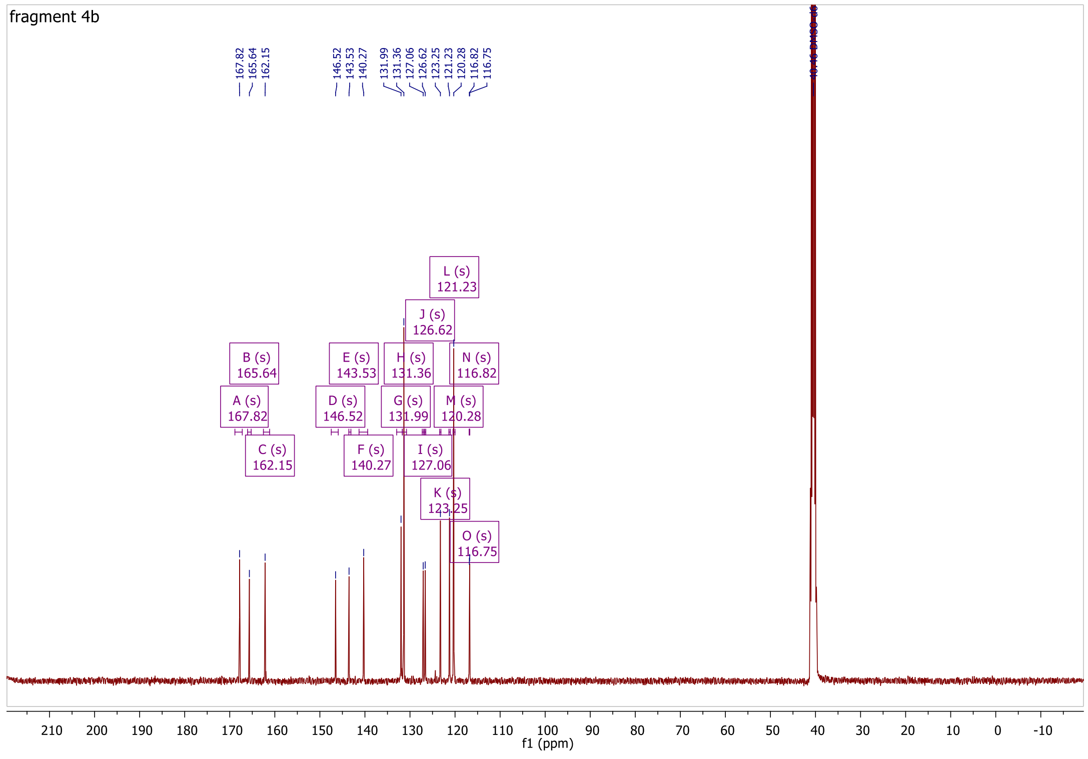

Fragment **4c**: ^{1}H NMR (400 MHz, DMSO-d_6)

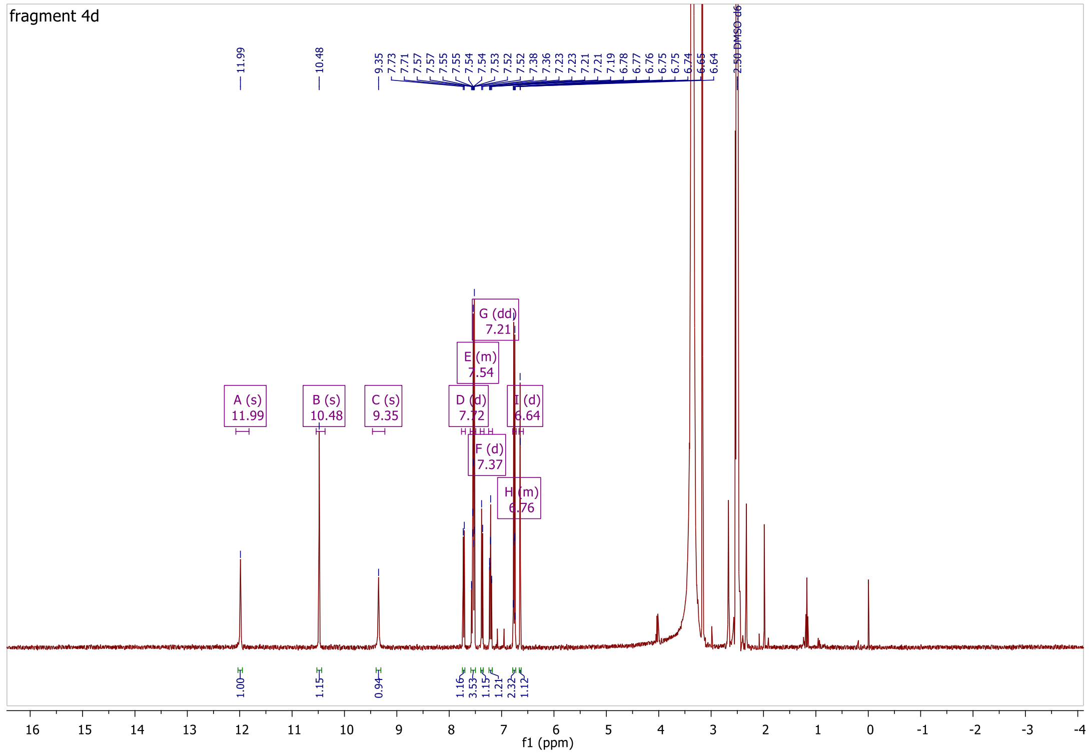

Fragment **4c**: ^{13}C NMR (101 MHz, DMSO-d_6)

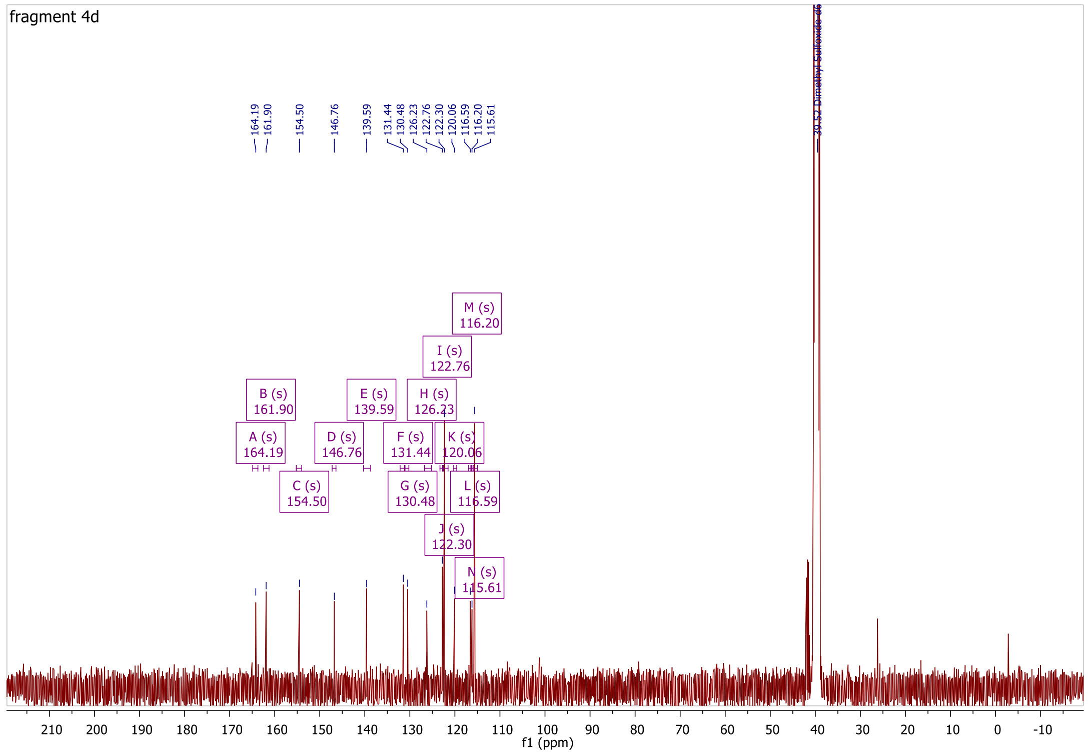

Compound **5a**: ^{1}H NMR (400 MHz, DMSO-d_6)

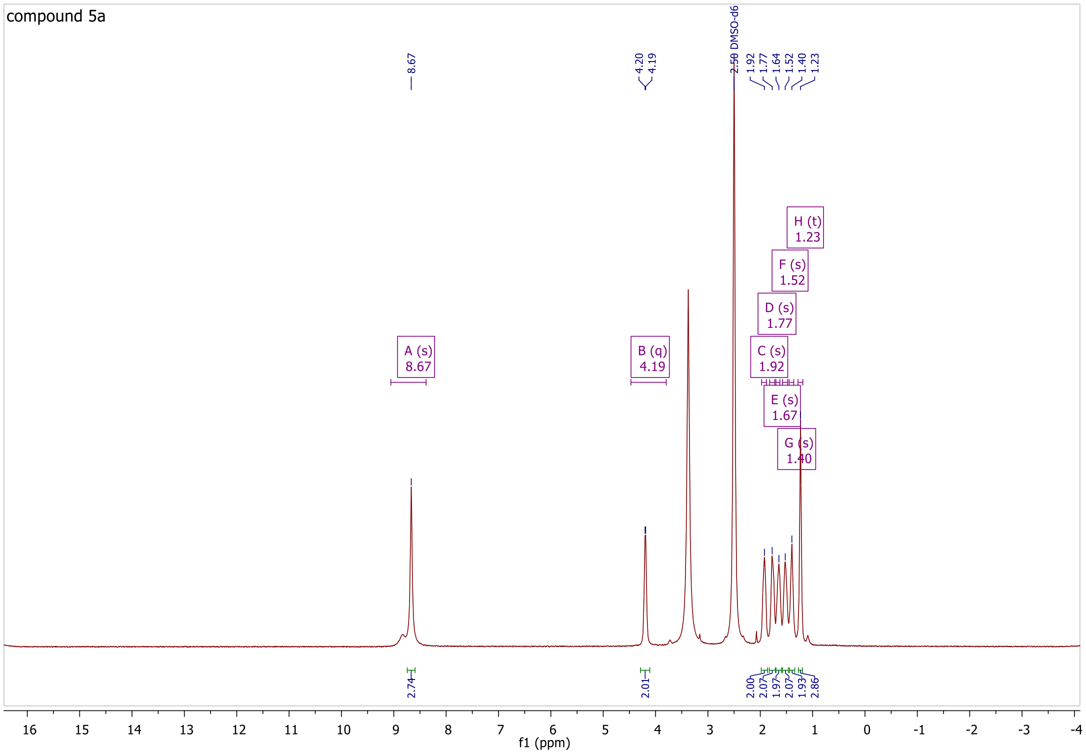

Compound **5b**: ^{1}H NMR (400 MHz, DMSO-d_6)

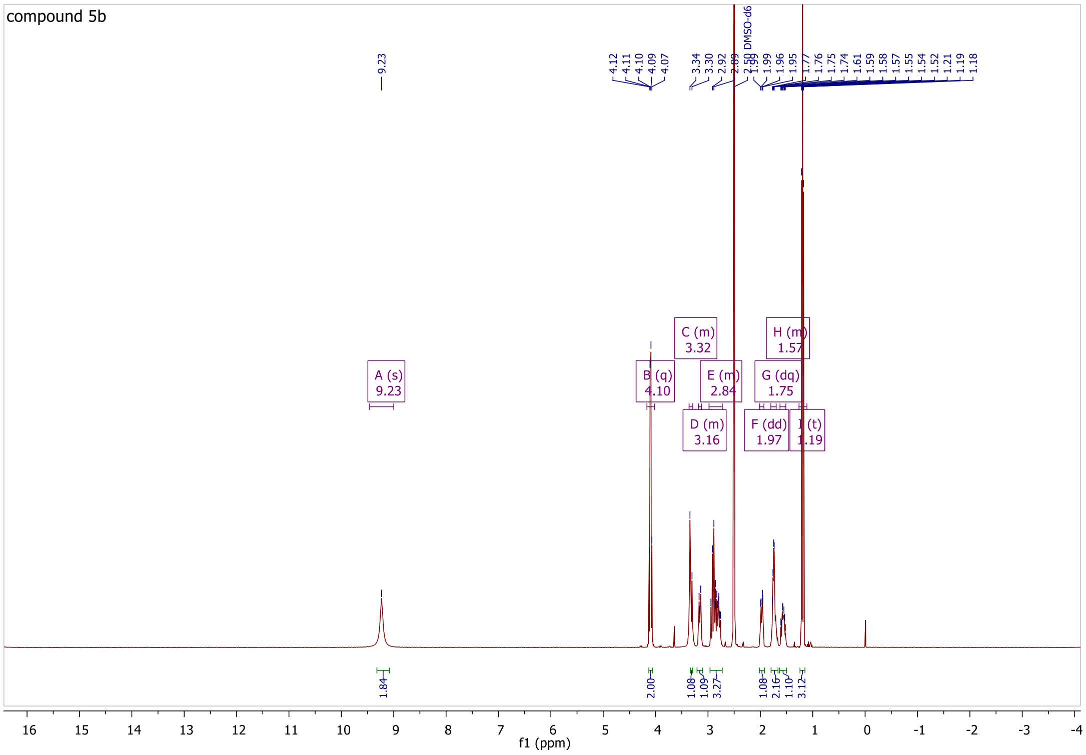

Compound **5c**: ^{1}H NMR (400 MHz, DMSO-d_6)

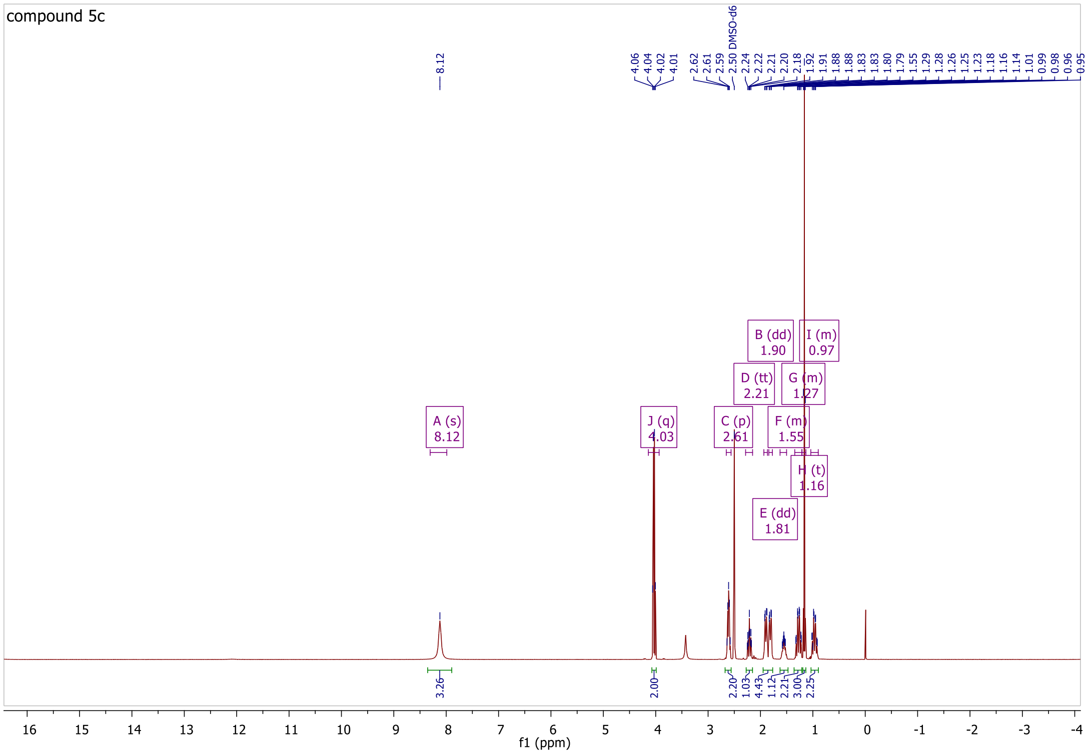

Compound **5d**: ^{1}H NMR (400 MHz, DMSO-d_6)

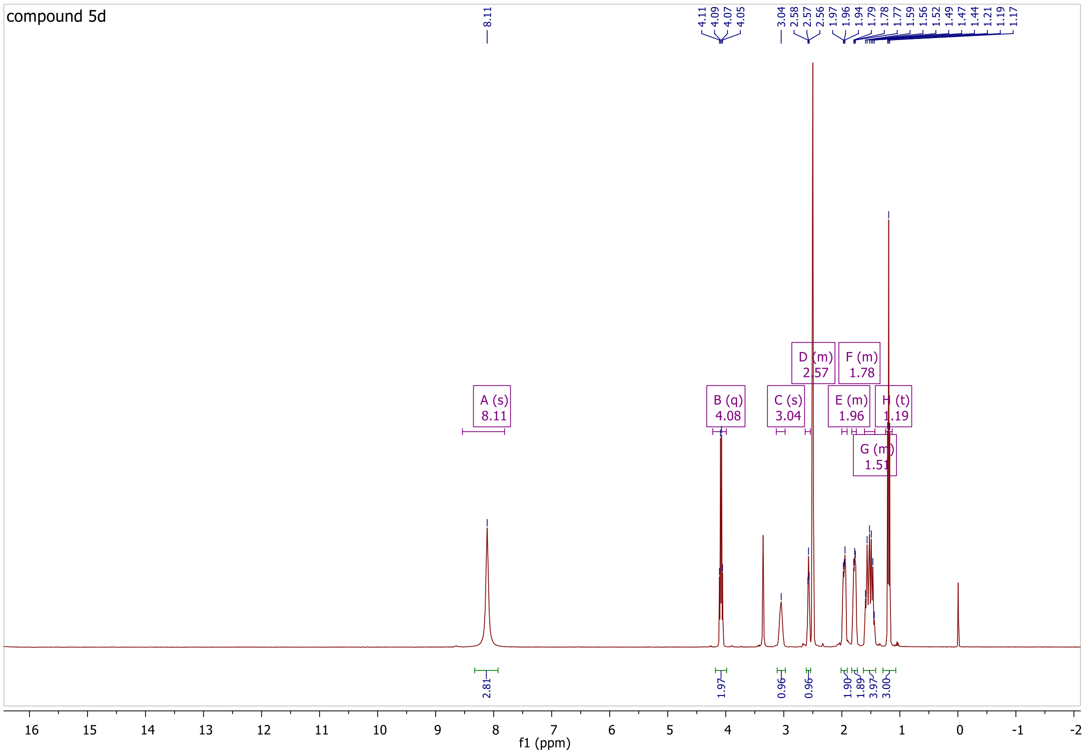

Compound **6a**: ^{1}H NMR (400 MHz, DMSO-d_6)

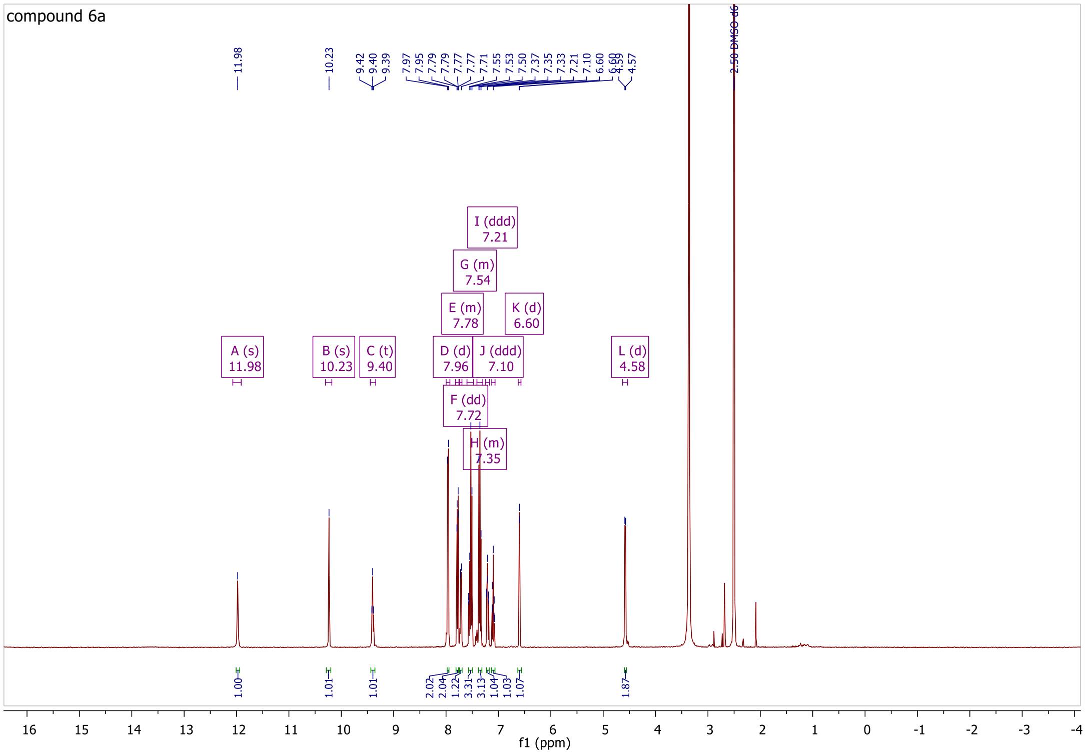

Compound **6a**: ^{13}C NMR (101 MHz, DMSO-d_6)

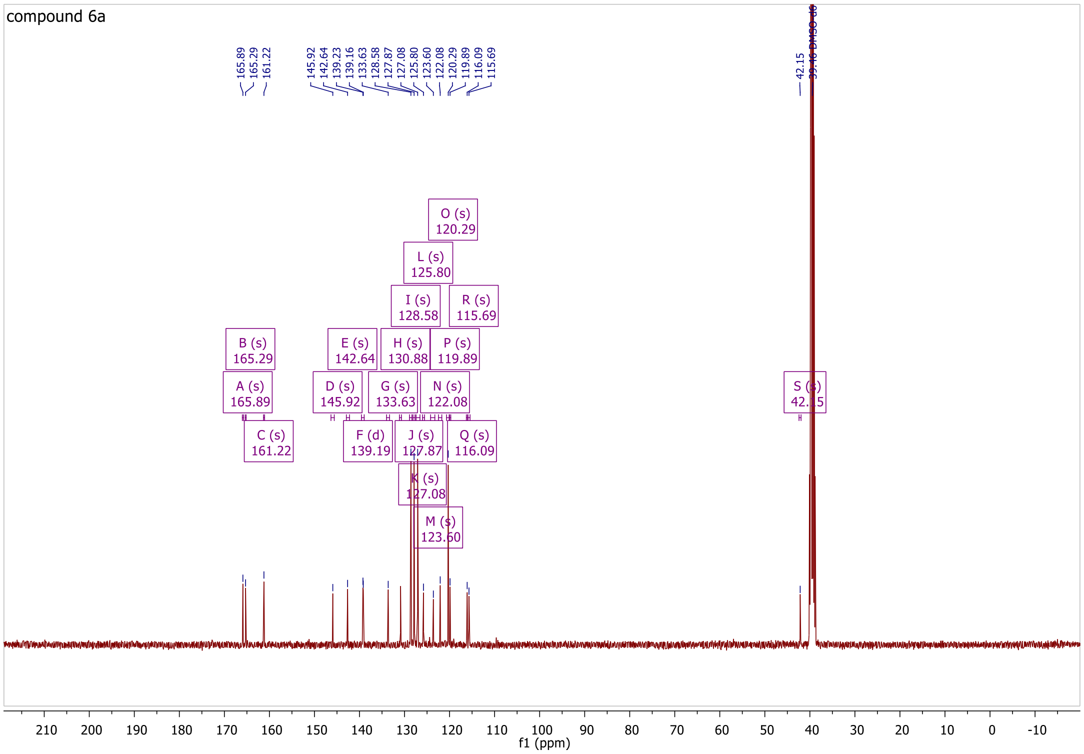

Compound **6b**: ^{1}H NMR (400 MHz, DMSO-d_6)

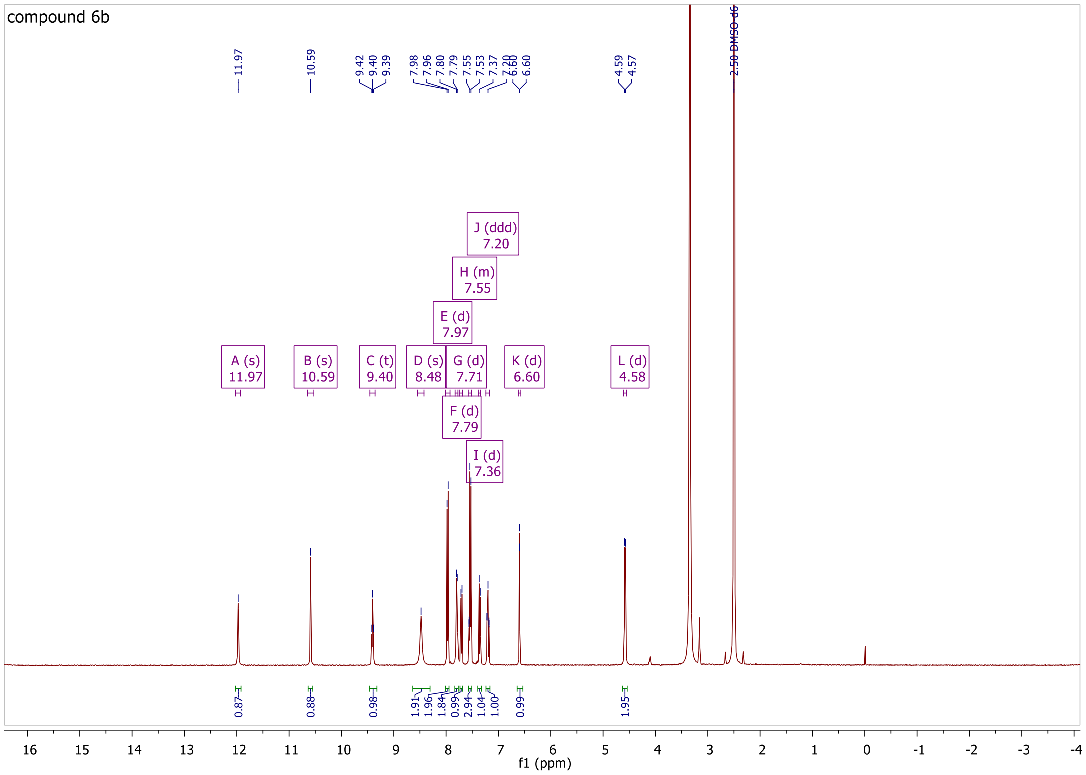

Compound **6b**: ^{13}C NMR (101 MHz, DMSO-d_6)

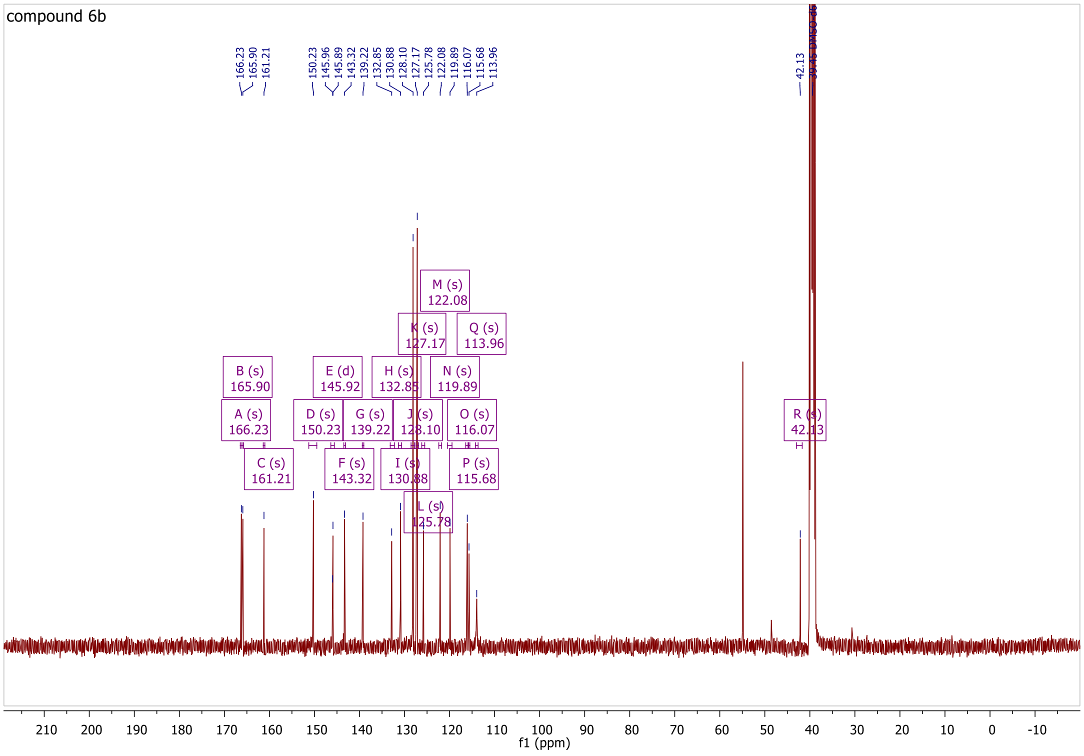

Compound **6c**: ^{1}H NMR (400 MHz, DMSO-d_6)

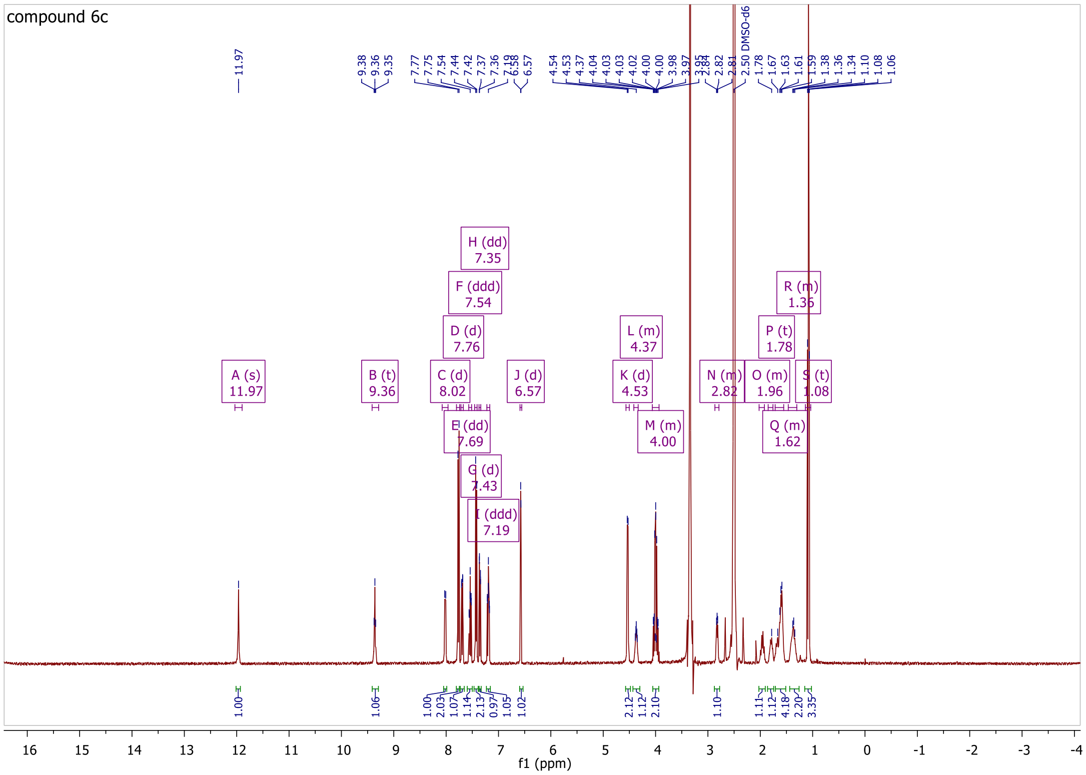

Compound **6c**: ^{13}C NMR (101 MHz, DMSO-d_6)

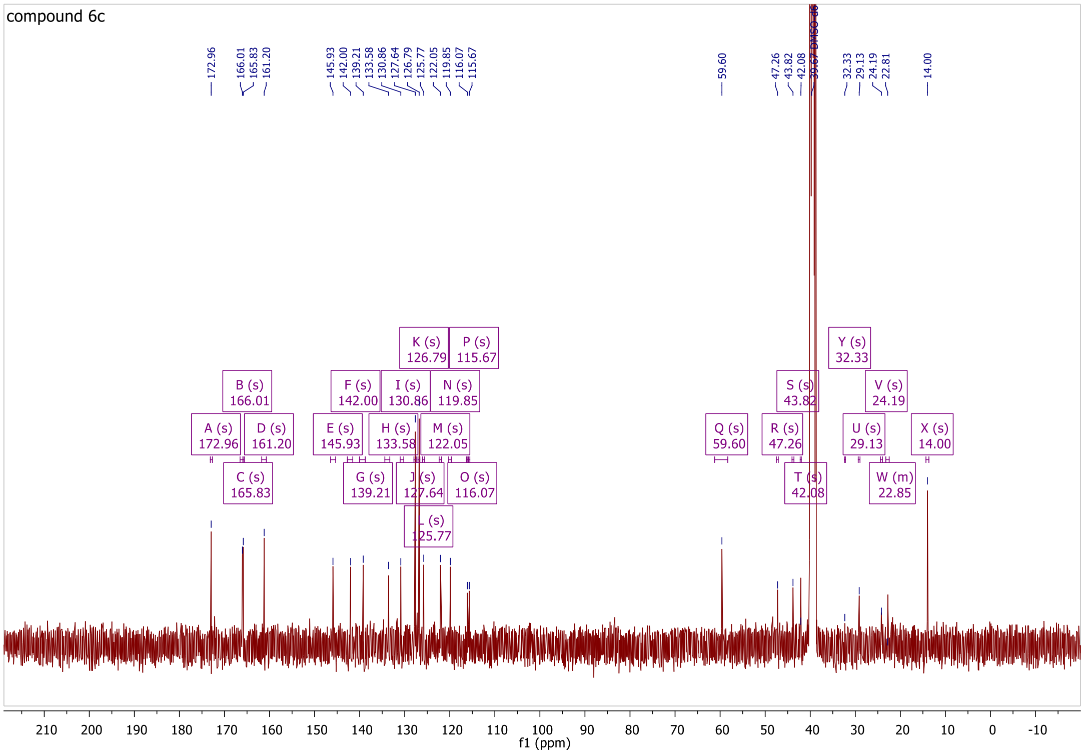

Compound **6d**: ^{1}H NMR (400 MHz, DMSO-d_6)

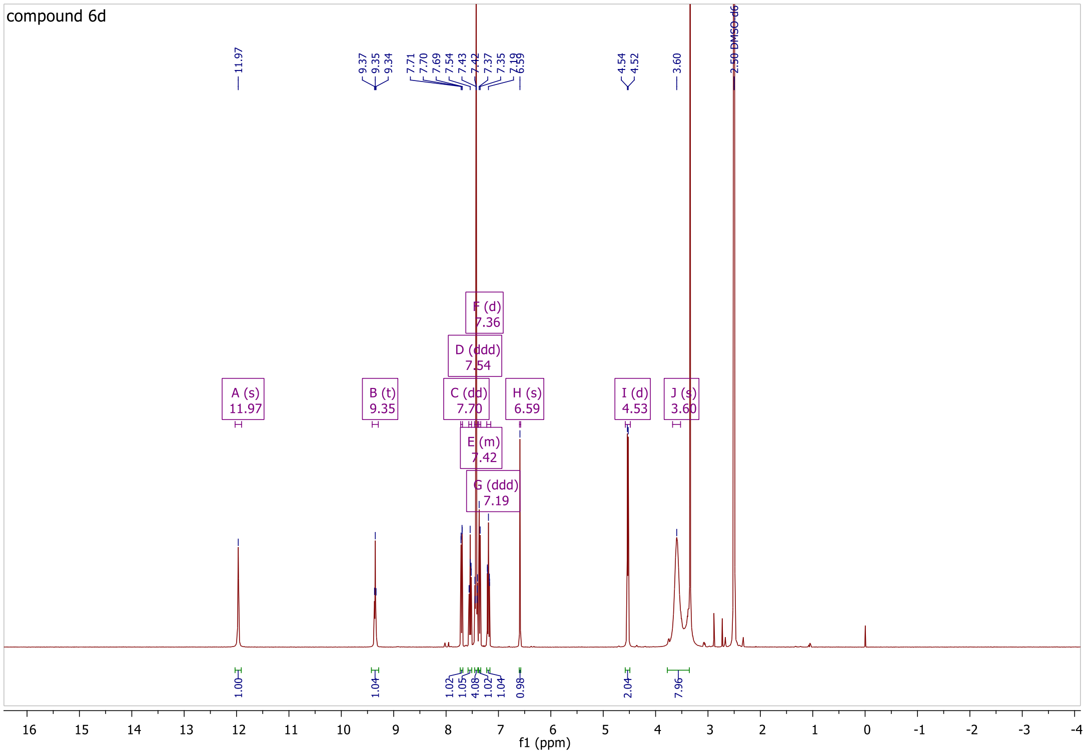

Compound **6d**: ^{13}C NMR (101 MHz, DMSO-d_6)

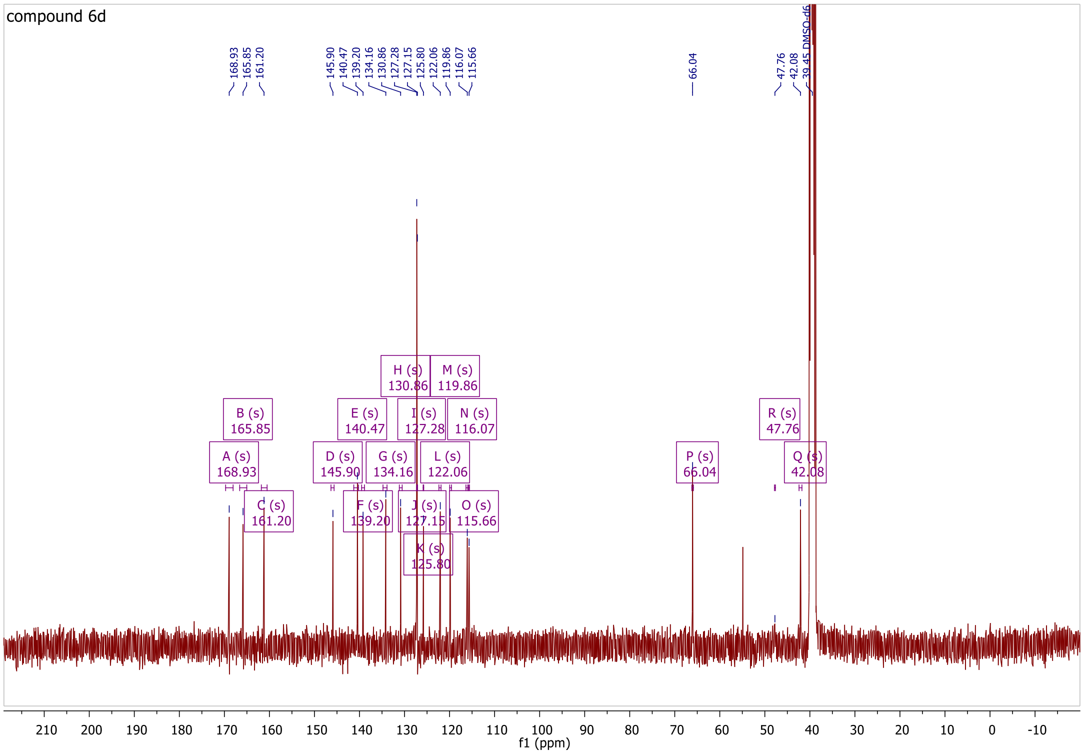

Compound **6e**: ^{1}H NMR (400 MHz, DMSO-d_6)

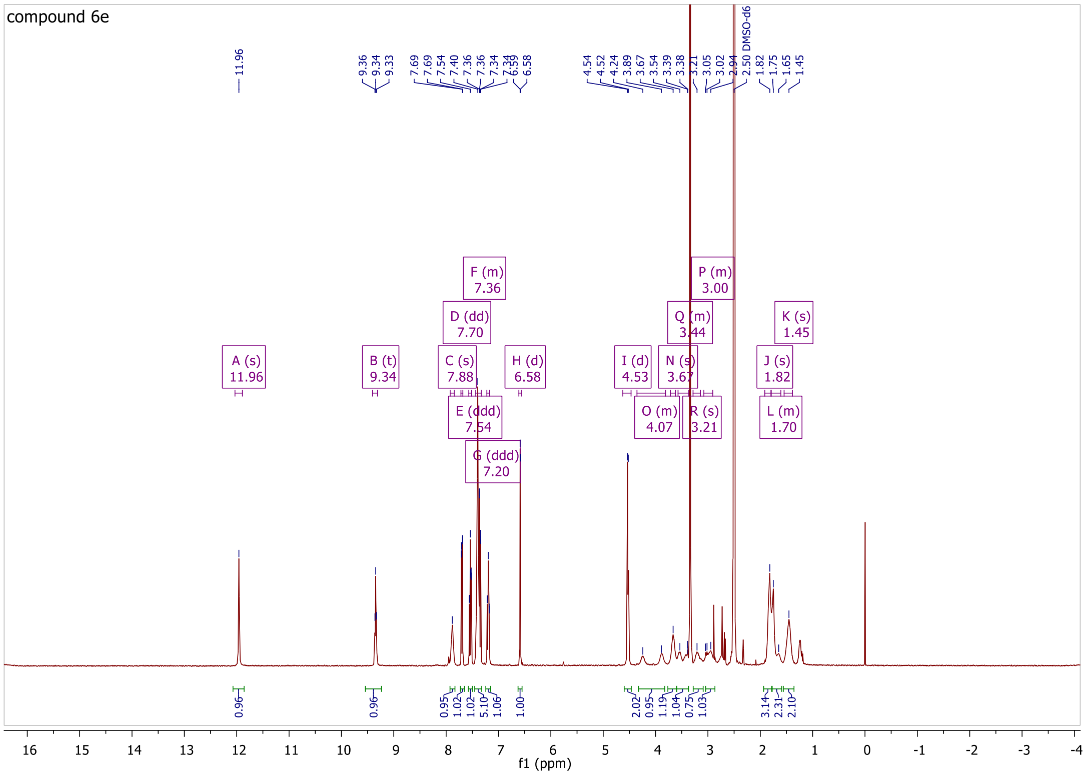

Compound **6e**: ^{13}C NMR (101 MHz, DMSO-d_6)

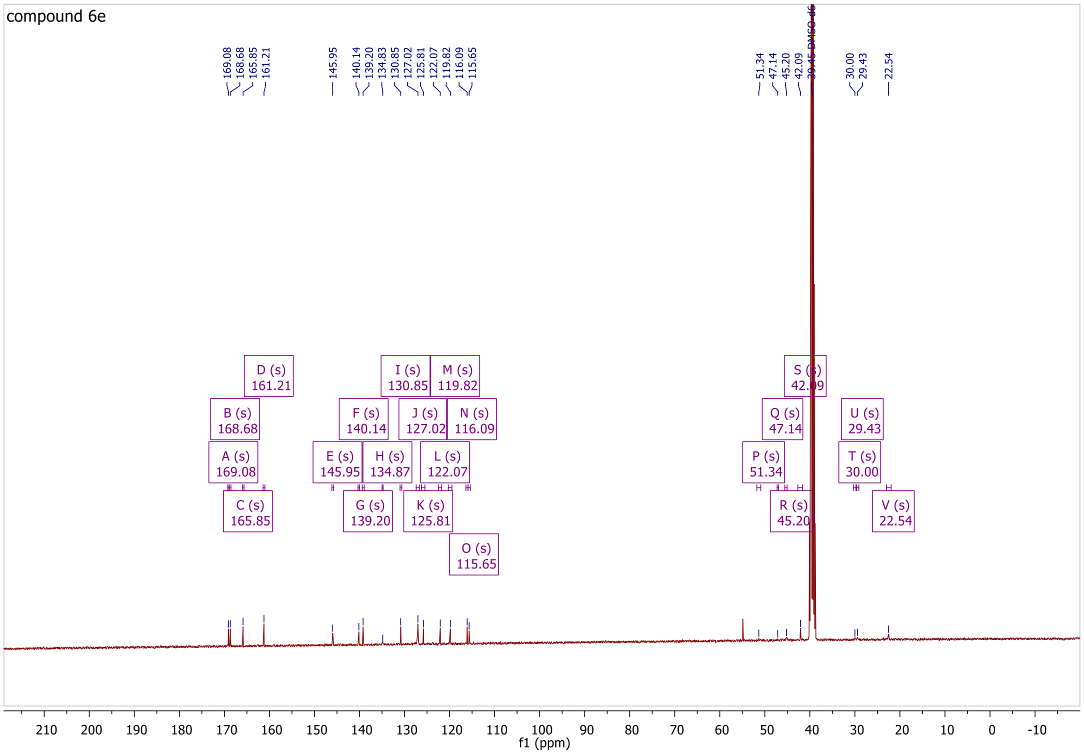

Compound **6f**: ^{1}H NMR (400 MHz, DMSO-d_6)

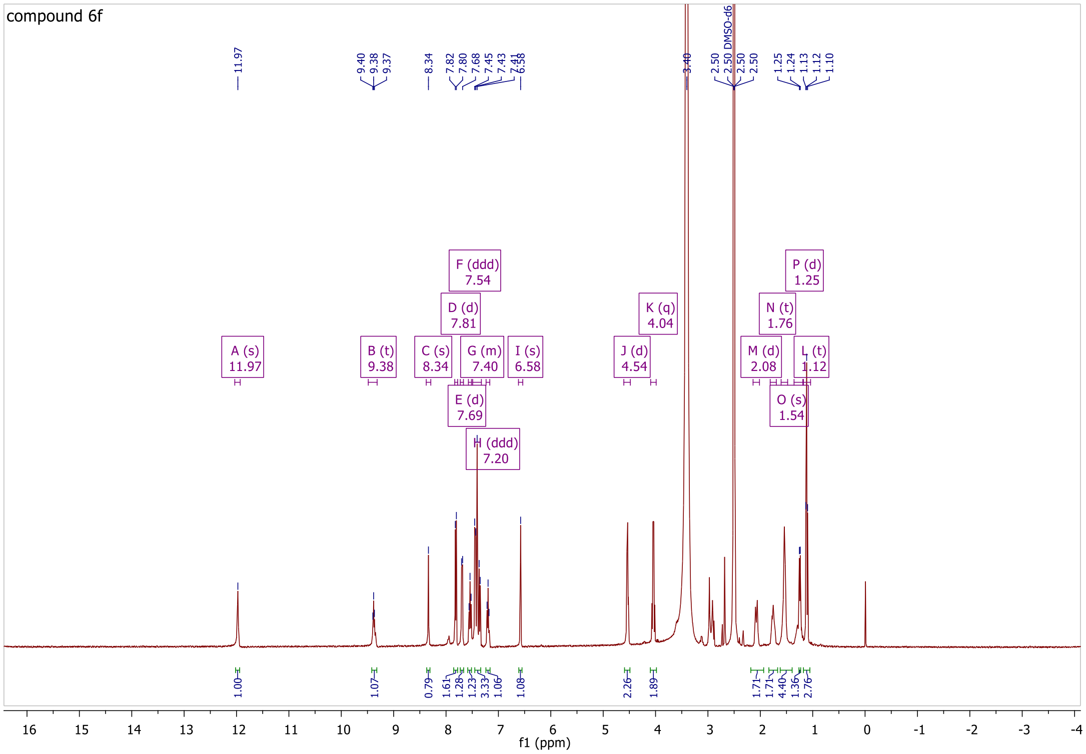

Compound **6f**: ^{13}C NMR (101 MHz, DMSO-d_6)

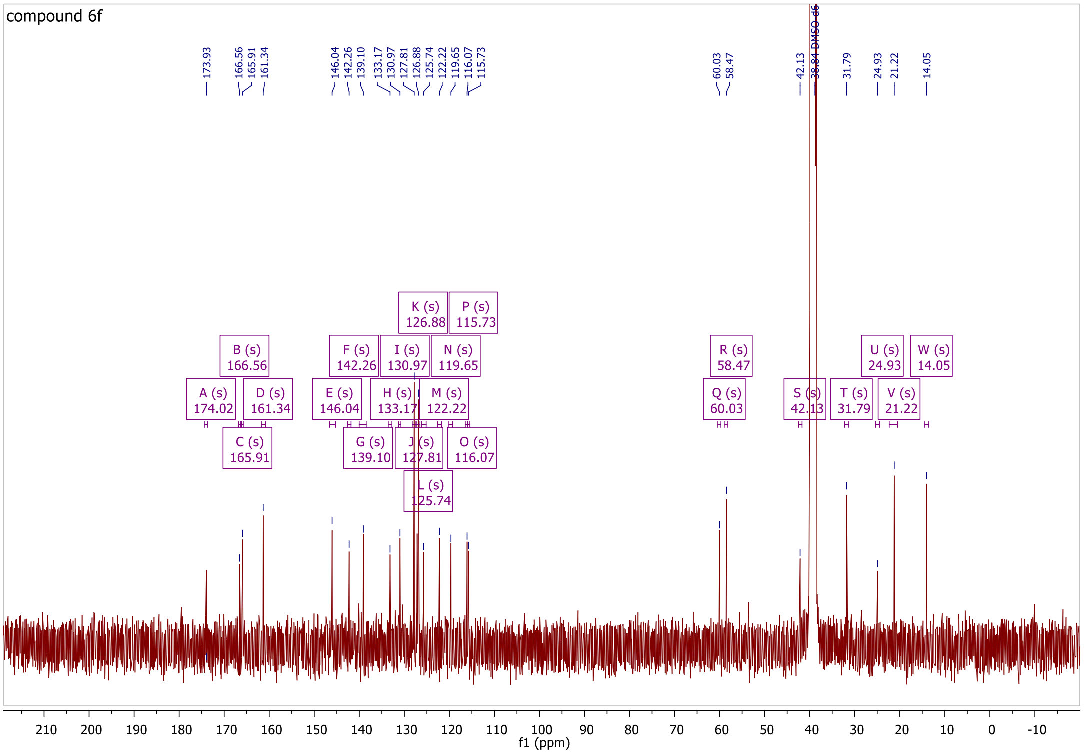

Compound **7a**: ^{1}H NMR (400 MHz, DMSO-d_6)

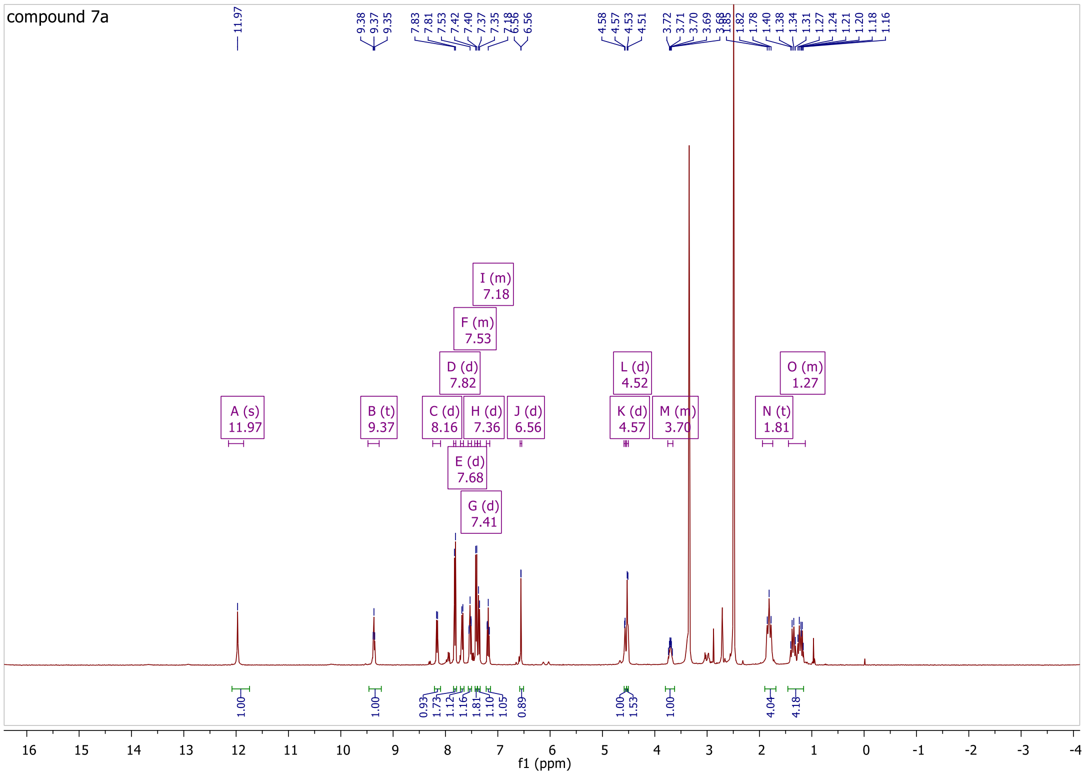

Compound **7a**: ^{13}C NMR (101 MHz, DMSO-d_6)

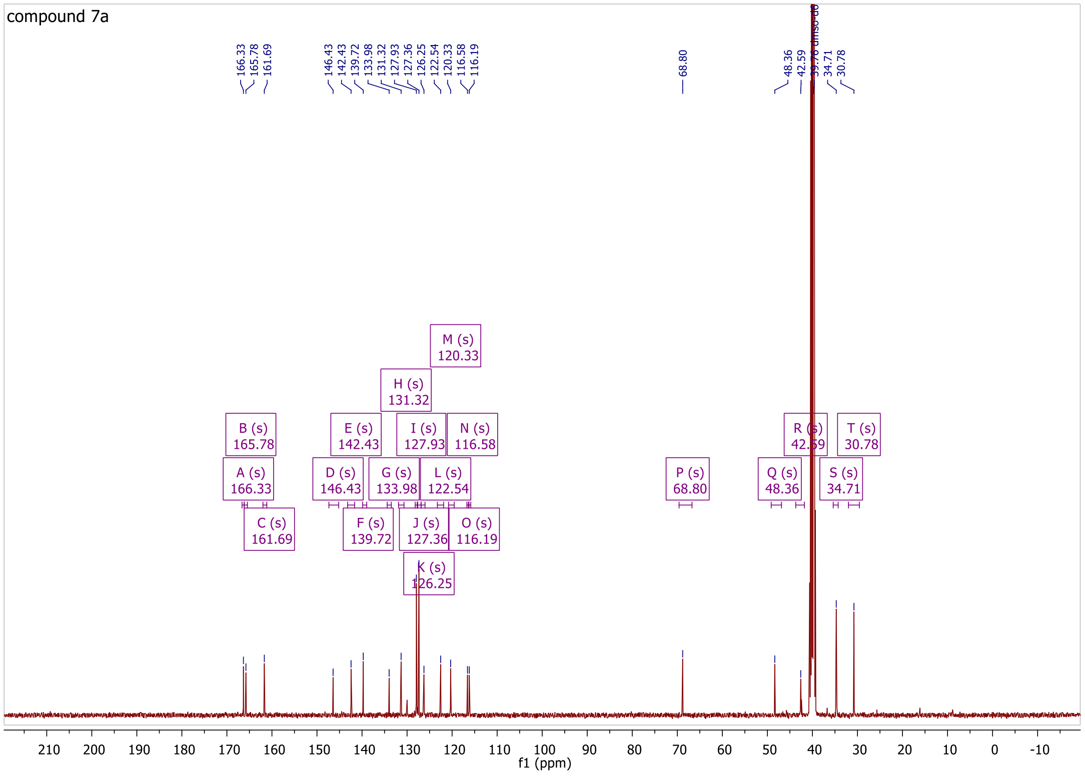

Compound **7b**: ^{1}H NMR (400 MHz, DMSO-d_6)

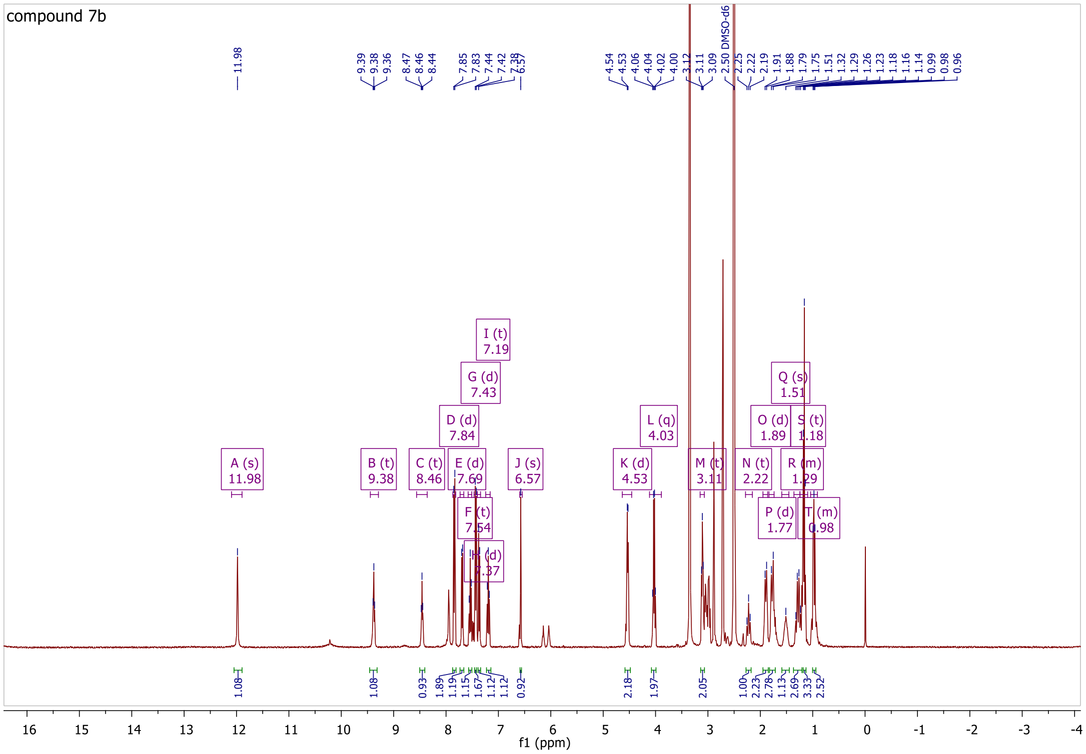

Compound **8a**: ^{1}H NMR (400 MHz, DMSO-d_6)

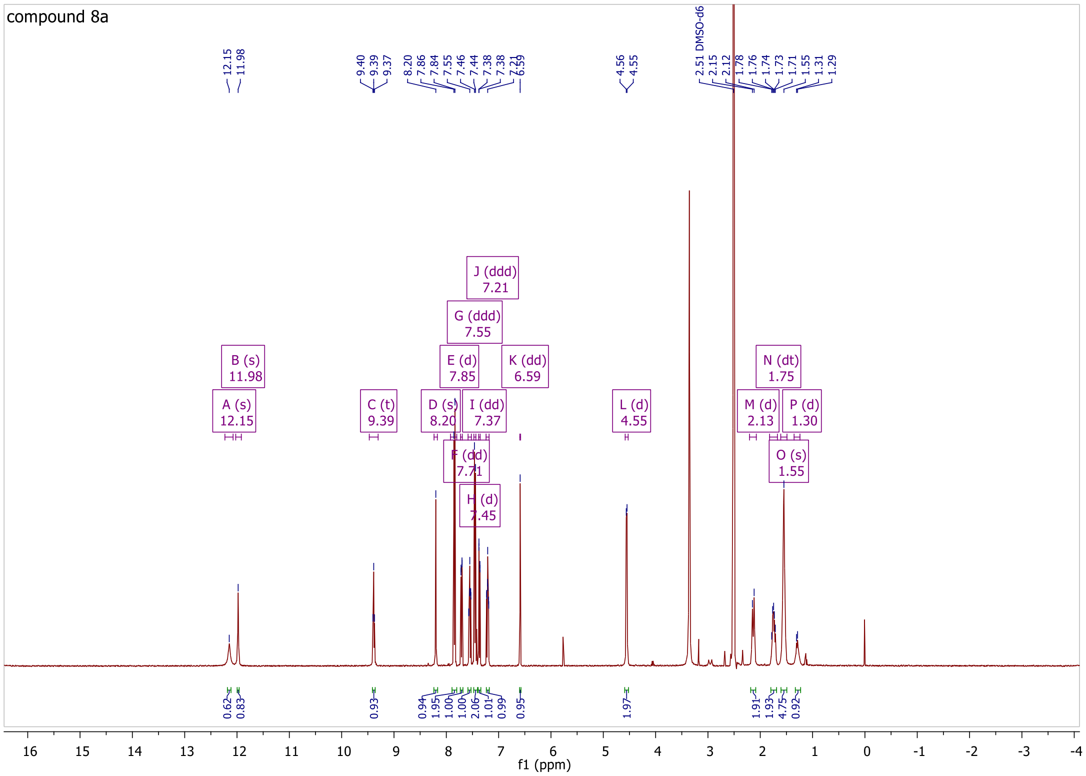

Compound **8a**: ^{13}C NMR (101 MHz, DMSO-d_6)

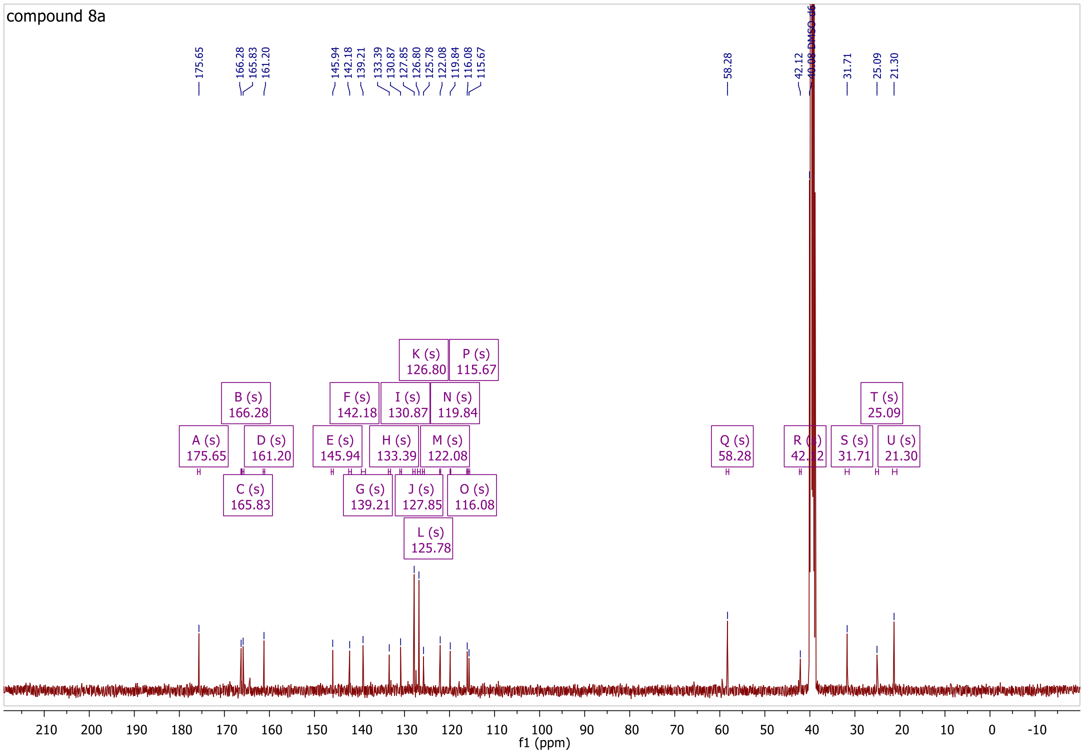

Compound **8b**: ^{1}H NMR (400 MHz, DMSO-d_6)

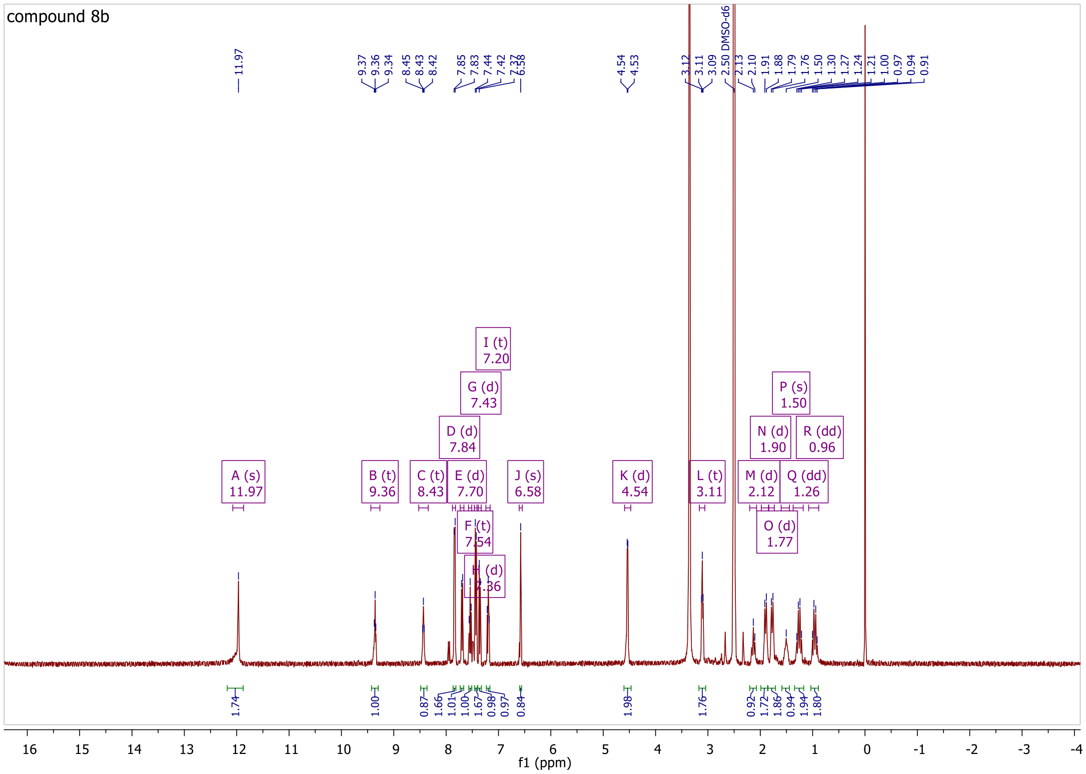

Compound **8b**: ^{13}C NMR (101 MHz, DMSO-d_6)

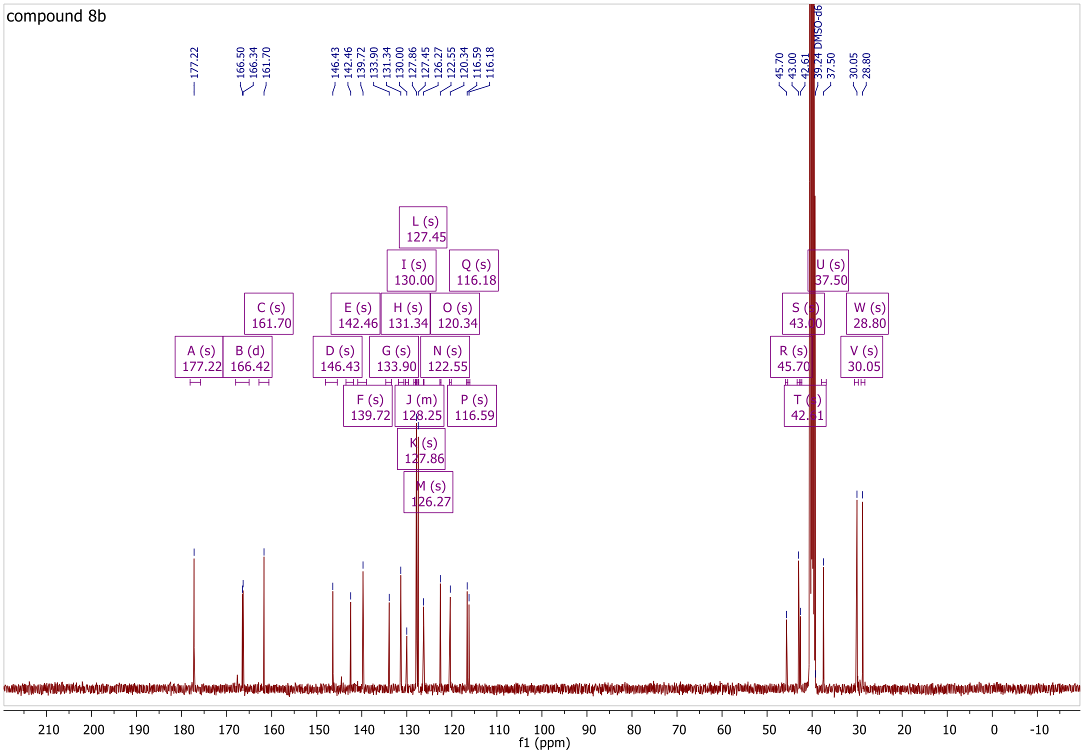

Compound **8c**: ^{1}H NMR (400 MHz, DMSO-d_6)

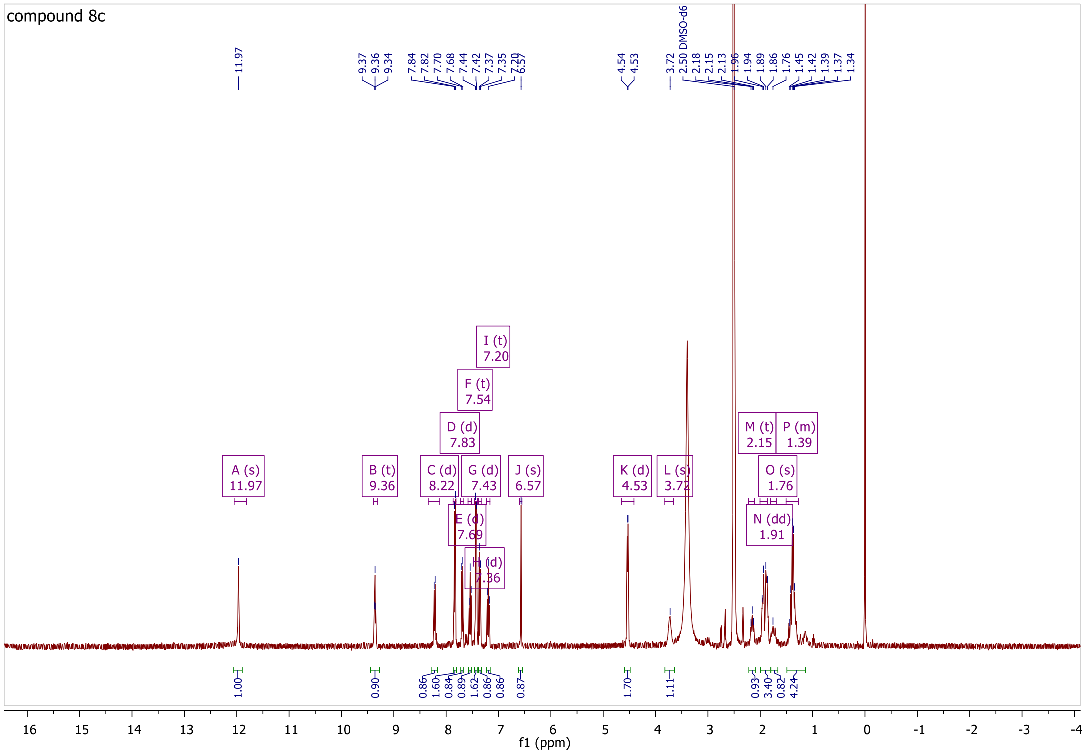

Compound **8c**: ^{13}C NMR (101 MHz, DMSO-d_6)

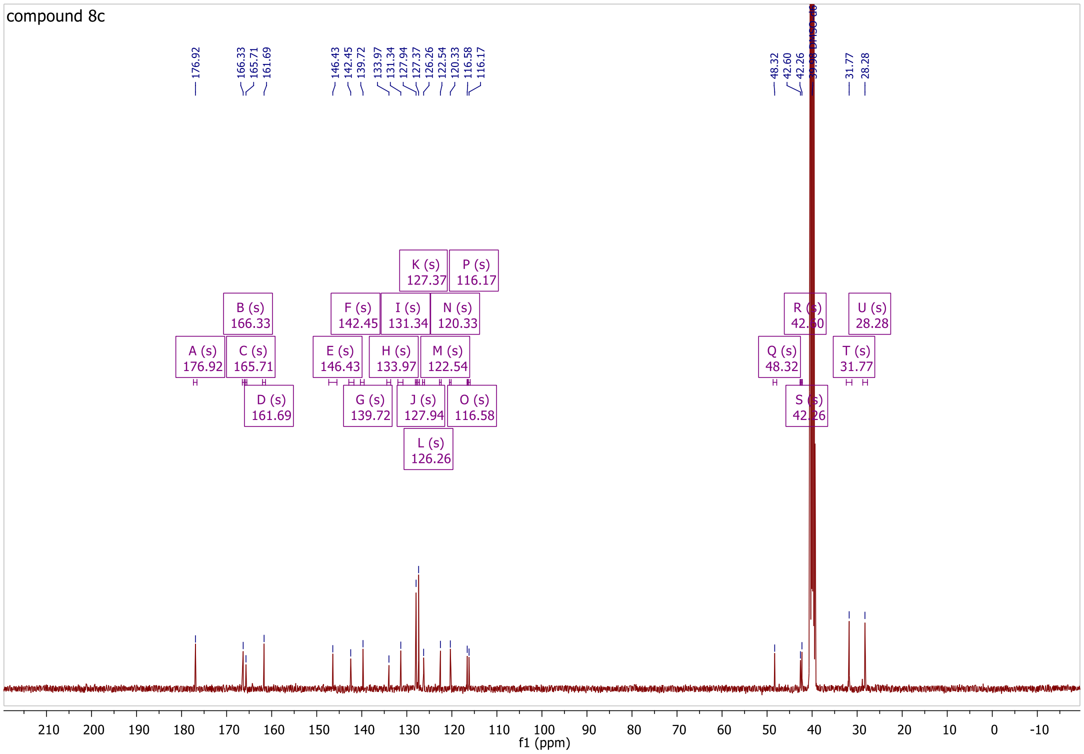

Compound **8d**: ^{1}H NMR (400 MHz, DMSO-d_6)

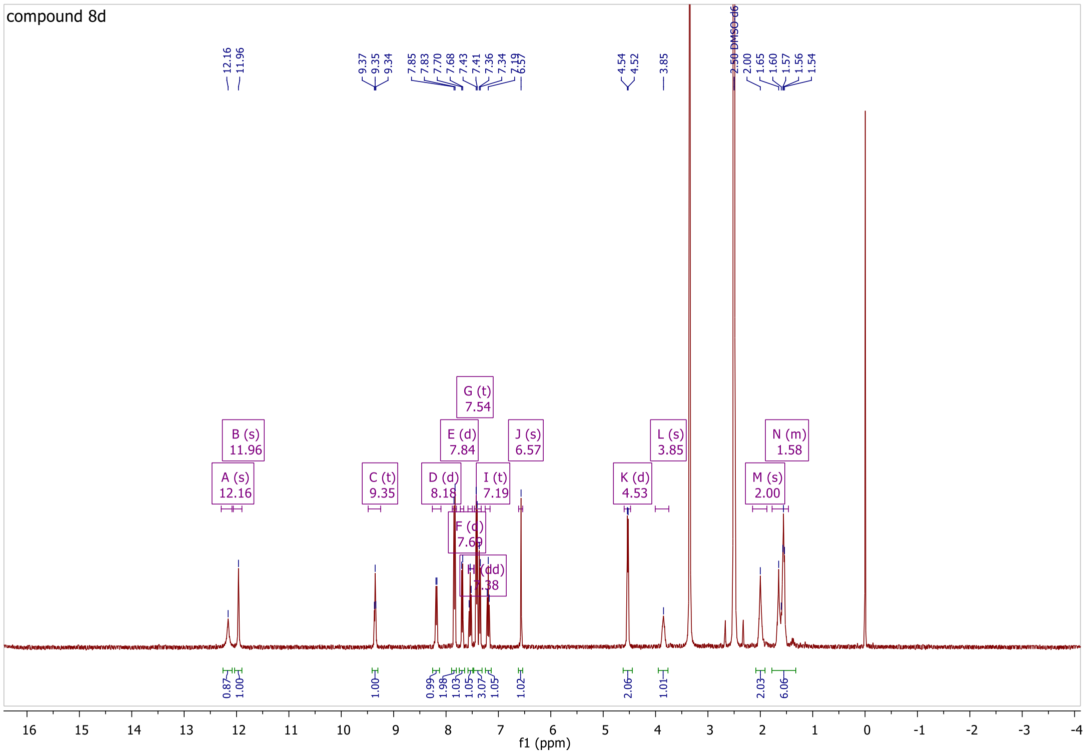

Compound **8d**: ^{13}C NMR (101 MHz, DMSO-d_6)

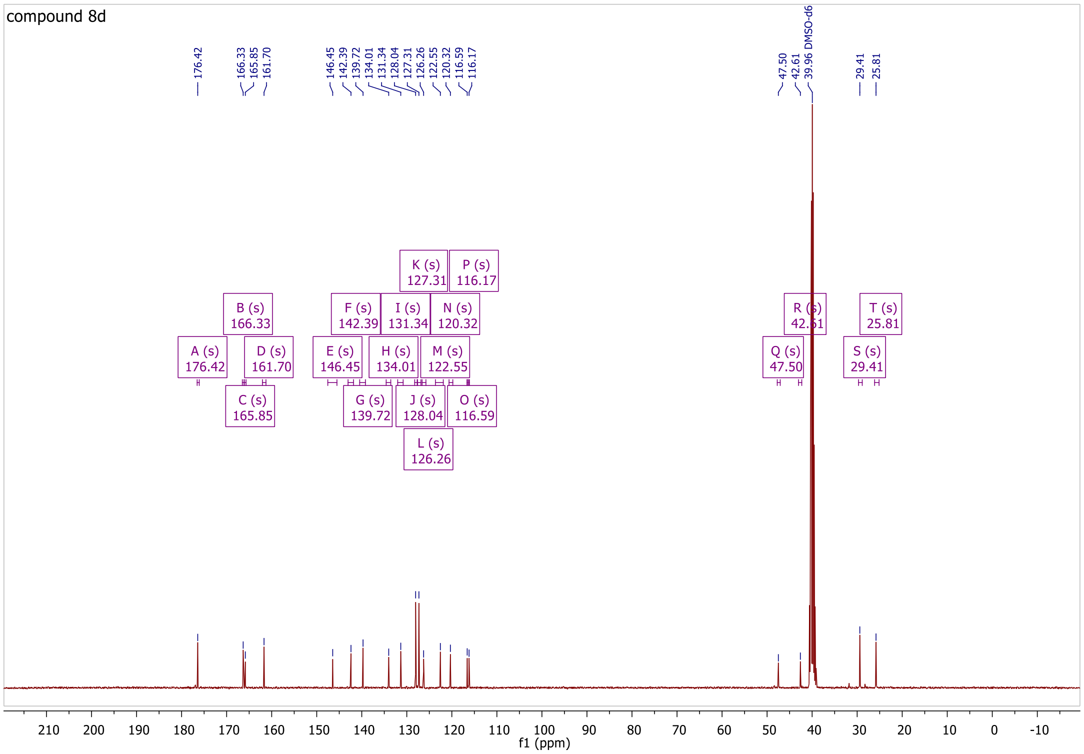

REFERENCES

Chhun, C., and Schmitzer, A.R. (2011). A Pseudorotaxane Umbrella Thread with Chloride Transmembrane Transport Properties. *Medchemcomm* 2 (10): 987–90. doi: 10.1039/c1md00128k.

Curry, J.E., Lyons, J.F., Squires, M.S., Thompson, N.T., Thompson, K.M., and Wyatt, P.G. (2006). Pharmaceutical Compounds. Patent Application Publication No. WO2006077424, World Intellectual Property Organization.

Dorogov, M. V., Ivanovsky, S.A., Khakhina, M.Y., Kravchenko, D. V., Tkachenko, S.E., and Ivachtchenko, A. V. (2006). Synthesis of 7-Sulfamoyl-Substituted 2-Oxo-2,3,4,5-Tetrahydro-1*H*-Benzo[b] Azepines. *Synth. Commun.* 36 (23): 3525–35. doi: 10.1080/00397910600943493.

Dulla, B., Wan, B., Franzblau, S.G., Kapavarapu, R., Reiser, O., Iqbal, J., et al. (2012). Construction and Functionalization of Fused Pyridine Ring Leading to Novel Compounds as Potential Antitubercular Agents. *Bioorganic Med. Chem. Lett.* 22 (14): 4629–35. doi: 10.1016/j.bmcl.2012.05.096.

Hogg, K.F., Trowbridge, A., Alvarez-Pérez, A., and Gaunt, M.J. (2017). The α-Tertiary Amine Motif Drives Remarkable Selectivity for Pd-Catalyzed Carbonylation of β-Methylene C-H Bonds. *Chem. Sci.* 8 (12): 8198–8203. doi: 10.1039/c7sc03876c.

Shonberg, J., Herenbrink, C.K., López, L., Christopoulos, A., Scammells, P.J., Capuano, B., et al. (2013). A Structure–Activity Analysis of Biased Agonism at the Dopamine D2 Receptor. *J. Med. Chem.* 56 (22): 9199–9221. doi: 10.1021/jm401318w.

Zhang, H., Tomašič, T., Shi, J., Weiss, M., Ruijtenbeek, R., Anderluh, M., et al. (2018). Inhibition of O-GlcNAc Transferase (OGT) by Peptidic Hybrids. *Medchemcomm* 9 (5). doi: 10.1039/c8md00115d.
